# Spray-dried hard carbon–Sn composites for energy-dense Na-ion batteries

**DOI:** 10.1039/d5eb00188a

**Published:** 2025-10-10

**Authors:** Giovanni Gammaitoni, Gihoon Cha, Rajkumar Reddy Kolan, Silke Christiansen, François Fauth, Matteo Bianchini

**Affiliations:** a Faculty of Biology, Chemistry and Earth Sciences, University of Bayreuth Universitätstraße 30 95447 Bayreuth Germany matteo.bianchini@uni-bayreuth.de; b Bavarian Center for Battery Technology (BayBatt) Weiherstraße 26 95448 Bayreuth Germany; c Fraunhofer Institute for Ceramic Technologies and Systems IKTS Äußere Nürnberger Straße 62 91301 Forchheim Germany; d CELLS-ALBA Synchrotron, Cerdanyola del Vallès 08290 Barcelona Spain

## Abstract

Sustainability and availability of raw materials, besides the usual performance-related metrics, have become crucial aspects for the development of new battery technologies to complement the existing successful Li-ion batteries. Sodium-ion batteries (SIBs) are at the forefront in this respect; however, the development of electrode materials achieving the expected performances is a challenge. Hard carbons (HC) are the most used anode material for SIBs, but poor gravimetric and volumetric capacities have limited the development of energy-dense SIBs. High-density and high-capacity metals that can react with sodium through formation/alloying reactions represent a possible solution, but the huge volume expansion during electrochemical cycling limits their utilization. In this work we explore the synthesis and characterization of sustainable hard carbon–Sn composites as anode materials for Na-ion batteries, with the aim of increasing HC performance without suffering the side effects of Sn volume expansion. Starting from conventional HC synthesis we propose a water-based continuous-flow spray-drying process to prepare our composites, resulting in an increase in HC's gravimetric and volumetric performances, including better long cycling stability. By using a set of analytical tools, we reveal the different physicochemical properties of our composites as a function of the starting cellulose precursors. The amount of Sn in the composites has been carefully evaluated through several techniques and lies at ≈15 or ≈25 wt% depending on the Sn content used for the synthesis. The activation of Sn during electrochemical discharge has been confirmed by *operando* synchrotron XRD, and the results show the appearance of sodiated Sn phases forming in kinetically driven reactions that do not fully adhere to the expected thermodynamic phase diagram. The electrochemical testing of our materials, carried out using conventional carbonate-based electrolytes, demonstrates excellent performances, with one composite demonstrating 301 mAh g^−1^ of capacity after 100 cycles (94% retention). Noteworthy is that the volumetric energy density is also significantly improved. Finally, by synthesizing several HC–Sn composites using alternative methods we demonstrate how spray drying leads to superior performances, especially in terms of capacity retention. Our work establishes the feasibility of spray drying as a scalable and sustainable synthesis route to prepare high-performance negative electrode composites for Na-ion batteries.

## Abstract 2

Volumetric and gravimetric capacity of hard carbon anodes is improved by synthesizing composites with tin by spray-drying. Structure, morphology and Sn content are widely investigated, as well as its activity during electrochemical cycling.

## Graphical abstract

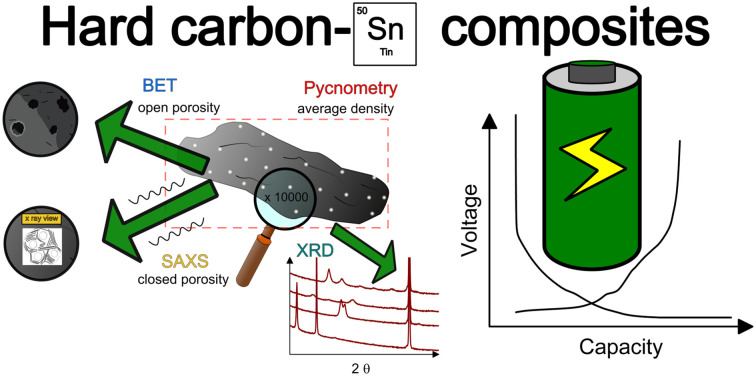

**Broader context**

Sodium-ion batteries (SIBs) are a novel technology that can complement Li-ion batteries (LIBs). SIBs would be strongly preferable in terms of the abundance of raw materials, cost and sustainability, yet they suffer from lower energy density, which ultimately hinders their widespread adoption. One of the causes of low energy density, specific and especially gravimetric, are the amorphous hard carbon (HC) anodes, which are required since graphite, used in LIBs, does not store sodium ions. A possible way to increase their energy density is to prepare composites of HC with metallic elements that alloy with sodium. A limited amount of such alloying elements can enable an increase of the capacity, without suffering from their main drawback, *i.e.* excessive volume expansion during electrochemical cycling. Here we report the reproducible preparation of HC–Sn composites using an aqueous, scalable spray-drying process. After discussing the physicochemical properties of the composites and quantifying in detail the Sn content, we demonstrate the Sn electrochemical activity. The cycling performances obtained indicate higher specific and especially volumetric capacity, as well as excellent long-term stability. The detailed analysis of our hard carbon–Sn composites can lead to further optimised materials, which will contribute to the advancement of sustainable sodium-ion technologies.

## Introduction

Sodium-ion batteries (SIBs) are widely investigated as an energy storage system able to offer alternative solutions for increasing energy storage demands.^1^ They were developed in the past century in parallel with lithium-ion batteries (LIBs),^2–4^ but the latter saw huge success and faster commercialization thanks to the lower molecular mass of Li and higher operating voltage of the cell, resulting in higher gravimetric and volumetric energy densities (Wh kg^−1^ and Wh L^−1^).^5^ However, the critical situation of lithium resources and its fluctuating price has pushed scientists to find alternatives for LIBs. The challenge lies in the fact that SIBs cannot provide the same performances as the best LIBs based on graphite and NCM electrodes; yet recently it was shown that a NVP/HC sodium-ion battery (Na_3_V_2_(PO_4_)_3_/hard carbon) could achieve a performance close to that of the LiFePO_4_/graphite (LFP/Gr) lithium-ion battery.^6^ NVPF/HC cells (Na_3_V_2_(PO_4_)_2_F_3_/hard carbon) have been commercialized by the French startup Tiamat,^7^ while several Chinese companies, such as CATL, offer layered oxide-based SIBs. The challenge is now to bridge the gap to LFP-based LIBs, to mitigate the expected huge demand for lithium in the next years.^8^ For both cells’ technology, a determinant limiting factor is the low density of the carbon anode, which reduces the overall volumetric energy density (Wh L^−1^). In LIBs, graphite can reach a density of 2.3 g cm^−3^, while hard carbons in SIBs typically have a lower density close to 1.5 g cm^−3^.^9^ Even though the specific gravimetric capacity of these materials is similar (372 *vs*. ≈300 mAh g^−1^), the lower density of HC contributes to the lower volumetric performances of SIBs. In the last years, scientists have tried to improve the volumetric energy density of LIBs and SIBs by using different carbon-based anodes, synthesized *via* innovative and cutting-edge methods and potentially achieving specific capacities well beyond the 300 mAh g^−1^ mark.^10,11^ However, these syntheses are often hard to scale-up beyond the laboratory scale.

A widely recognized solution to increase the performance of the anode is to replace the carbon-based materials with elements that have the ability to alloy with Na, *e.g.* Sn, Ge, Sb, P, and Pb.^12–16^ These exhibit high densities and hence high specific gravimetric capacities (up to 10^3^ mAh g^−1^) when high sodium content phases are formed during electrochemical discharge.^15,17^ Special attention has been paid to Sn,^18,19^ since it shows a high theoretical gravimetric capacity (847 mAh g^−1^), a high density (7.31 g cm^−3^), and it is relatively abundant and non-toxic. However, as is well-known from the effect of Si on LIBs, these materials suffer from huge volume expansion during the sodiation process,^20^ leading to pulverization of the electrode and a short cell lifetime. Moreover, it is important to note that most of the synthesis methods reported in the literature involve special steps that are often applicable only to a laboratory scale synthesis, *e.g.* chemical vapour deposition (CVD), ball milling, the use of templates and solvothermal treatments.^16^ To mitigate the issue of excessive volume expansion, the scientific community focussed on preparing blends or composites of carbon and alloying elements. The first attempts were made for LIBs,^21–25^ and later for SIBs,^26–28^ but again many proposed syntheses are not scalable. Nonetheless in many commercialized LIBs today a blend of graphite and silicon constitutes the anode, but only ≈10 weight % of silicon is included in the composite to avoid excessive volumetric expansion and prevent electrode pulverization.

A promising scalable synthesis method for the production of composites is spray drying, which has been demonstrated in the preparation of anodes for LIBs and SIBs.^29,30^ A recent comprehensive study of alloying elements for SIBs proposed not to exceed the volume expansion that is found in commercial graphite/Si blends, which can be achieved by limiting the alloy weight % in the hypothetical HC/alloying-element blend in the SIB anode.^31^ It has been also demonstrated that ether-based electrolytes can mitigate the pulverization process by stabilizing the solid electrolyte interphase (SEI) that grows homogeneously on the surface of the anode and does not become too thick (compared to its evolution in commercial carbonate-based electrolytes).^32,33^ However, ether-based electrolytes are typically unstable at high voltages, and more in-depth research is required on the optics of combining optimized anodes and high voltage cathodes to assemble full cells.^34^ Therefore, the most used electrolytes remain carbonate-based ones that perform well at high voltages and represent a simple drop-in solution taken from LIB technology.

For these reasons, in this work we explore ways to increase the performance of HC *via* the incorporation of Sn using spray drying as a green and scalable method. Spray drying allows for continuous synthesis with a product yield that only depends on the amount of reactants, with no facility restrictions. We synthesized HC–Sn composites and compared their performances and characteristics to those of pristine HC. The morphology and microstructures are studied to understand the modifications induced by the spray-drying synthesis, and the cell performances are recorded in electrochemical cycling tests. Using a carbonate-based electrolyte, we demonstrate improvements in the HC–Sn composite performances, also comparing spray drying to other methods. Furthermore, we clarify the functioning mechanism of the composite.

## Experimental section

### Synthesis of materials

Hard carbons (HCs) were synthesized by a two-step heat treatment: we used two different cellulose precursors from Sigma-Aldrich, one was colloidal (microcrystalline) here named CC, and the other one was composed of fibers (microcrystalline powder 20 μm), here named FC. They are initially treated in a Linn furnace under a synthetic air atmosphere for 12 h at 300 °C, and then the final carbonization step takes place in a Carbolite furnace at 1400 °C for 1 h. The heating rate is 5 °C min^−1^ and then the samples are naturally cooled down. Then, HC and Sn nanoparticles (tin nanopowder 99.9%, 60–80 nm, US Research Nanomaterials) are used as precursors for the synthesis of HC–Sn composites (HC : Sn = 2.34 : 1). We used a Buchi spray dryer (Mini spray dryer B-290), and we set the inner temperature to 180 °C, the pumping rate to 30% and the aspirator to 100%. The synthesis happens in air. We labelled the composites as CC-Sn and FC-Sn, named after the HC used for the synthesis. Two more composites were prepared with target HC : Sn ratios equal to 1, and they are labelled CC-Sn_50 and FC-Sn_50. The effect of the spray-drying process on HCs was also determined by repeating the spray-drying synthesis without Sn nanoparticles. Two reference pairs of samples were synthesized to compare the performance of spray-dried samples; the first synthesis involved hand mixing of HCs and Sn nanoparticles in a mortar for 10 min, while the second synthesis was done in a planetary ball mill (Fritsch, Pulverisette 7) for 6 h at 600 rpm (balls : AM wt ratio equal to 20).

### Material characterization methods

Powder X-ray diffraction (XRD) patterns were collected at the MSPD beamline (ALBA synchrotron) with a position sensitive detector, MYTHEN2 (wavelength *λ* = 0.62020 Å), and in the laboratory using a Stoe StadiP diffractometer with a Mythen2K detector in Debye–Scherrer geometry equipped with monochromatic Mo-Kα_1_ radiation (wavelength *λ* = 0.70932 Å). Acquisition was performed on powder packed in a 0.5 mm diameter borosilicate glass capillary. Then we performed the Rietveld refinement using the FullProf Suite. The *operando* synchrotron X-ray powder diffraction (SXRPD) was done at the same beamline on coin cells equipped with a quartz window, in the half cell configuration. The experiment was done at 50 °C in a coin cell chamber where thermalization was guaranteed by the continuous circulation of a silicon oil, as carefully described in the literature.^35^ Patterns were recorded every 10 min with an effective integration time of 20 s.

TGA experiments were performed on a simultaneous thermal analyser (STA), model STA 449 F5 “Jupiter” (Netzsch), connected to a mass spectrometer for evolved gas analysis (QMS Aëolos Quadro model 8QMS403Q), with a protocol that involved heating to 600 °C at a rate of 5 °C min^−1^ and held at that temperature for 3 h, followed by natural cooling. CO_2_ evolution was monitored by following the *m*/*z* = 44 channel.

For Raman spectroscopy the samples were pelletized under a pressure of 0.5 MPa. The measurements were carried out with a WITEC ALPHA 300 RA+ instrument at the “Synthesis and Molecular Characterization” Keylab of the University of Bayreuth, using a wavelength of *λ* = 532 nm and a power of *ca.* 7.0 mW. The spectra were taken with a number of accumulations of 50 and an integration time of 0.5 s.

Density measurements were carried out with two pycnometer instruments: a gas pycnometer (Anton Paar Ultrapyc 5000 micro) and a glass pycnometer in combination with xylene (DIN 51901:1980-12). For the former, the volume of the material was determined by 15 runs with He gas. For the latter, the density of HCs has been determined knowing the density of xylene (0.865 g/cm^3^) and the total volume of the pycnometer (5.078 mL) and calculating the density of the material of interest by measuring the weight of the pycnometer filled with a known amount of xylene and material.

SEM and *ex situ* SEM-EDX was performed with a ThermoFischer Scientific Phenom ProX microscope on powder samples, with magnifications of 5000× and 1000×, an acceleration voltage of 15 kV and BSD and SED detectors. Before conducting the *ex situ* measurements, the samples were carefully washed with DMC and dried under vacuum. For electrode samples a LEO-1530 electron microscope was used at the “Electron and optical microscopy” Keylab of the University of Bayreuth, with a magnification of 500×, an acceleration voltage of 3 kV and an ESB detector.

SAXS patterns were collected at the “Mesoscale characterization: scattering techniques” Keylab of the University of Bayreuth over a *q* range of 0.001–1 Å^−1^. The acquisition was performed on powder packed in a 0.3 mm diameter borosilicate glass capillary. The measurements were performed in transmission geometry using a Double Ganesha AIR system (SAXSLAB) with a copper anode as the X-ray source (wavelength *λ* = 1.5406 Å) and a PILATUS 300 K, Dectris detector. For data treatment, the empty capillary was subtracted as the blank. The data were fitted with SASfit software.

The BET surface area was determined from N_2_ absorption experiments on a Quantachrome Autosorb 1 instrument at the Chair of Inorganic Colloids for Electrochemical Energy Storage of the University of Bayreuth. All samples were carefully outgassed at 150 °C under ultra-high vacuum overnight prior to measurements and liquid N_2_ was used to cool down the system for the measurement. The quenched solid density functional theory (QSDFT) method was used to determine the pore size distributions and the pore widths.^36^ The model employed takes into account the adsorption branch of N_2_ at 77 K on carbon considering the slit/cylindrical shape of the pores.

The porosity and volumetric composition of the electrode components, including hard carbon, Sn, and binder plus conductive carbon were analyzed using X-ray microscopy (XRM). Imaging experiments were performed with a Zeiss Xradia 620 Versa instrument (Carl Zeiss X-ray Microscopy Inc., Pleasanton, CA, USA). This system employs a cone-beam X-ray source and utilizes geometric and optical magnification to achieve high spatial resolution. The X-ray source generates a tunable polychromatic beam with a tungsten target, where the acceleration voltage can be adjusted to optimize imaging conditions. The XRM scans were conducted using a 20× objective lens to achieve high spatial resolution, with the source operated at a voltage of 50 kV and a power of 4.5 W. The imaging conditions included an air filter, an exposure per frame of 0.7 s, and 44 frames in total, enabling high-contrast volumetric data acquisition. Electrode samples with a diameter of 13 mm were carefully mounted onto a sharpened mechanical pencil lead and fixed into a custom-designed homemade holder to ensure stability during imaging while minimizing motion artifacts. The acquired XRM projection images were reconstructed using proprietary software (Zeiss XMReconstructor, Carl Zeiss X-ray Microscopy Inc., Pleasanton, CA, USA) with a filtered back-projection algorithm, and post-processing and visualization were conducted using XamFlow (Lucid Concepts AG, Zürich, Switzerland). The volumetric images obtained were utilized to determine the porosity and volumetric fractions of the electrode components by applying simple thresholding techniques for the identification and segmentation of key materials. The volume occupied by each component was calculated within a cubic volume of 0.00018 cm^3^ (120 μm × 1000 μm × 1500 μm) using the XamFlow workflow tool.

### Electrochemical measurements

Anodes were prepared by wet casting on Al foil. For slurry preparation we used different strategies depending on the active material used. For the HCs, we mixed the active materials with the binder in a ratio of 95 : 5, while for the HC–Sn composites we also added conductive carbon (Super P, Alfa Aesar, 99%), resulting in a ratio of 70 : 10 : 20. For the design of HC electrodes, there are different studies in the literature where several combinations of binders are tested.^37,38^ In this work we tested PVdF and a mixture of CMC : SBR with our materials and we found that the optimal binder differed depending on the morphology of the HC. Initially, two 7.5% solutions of binder dissolved in the solvent were prepared: we usedPVdF in NMP (*N*-methyl-2-pyrrolidone, Sigma-Aldrich, 99.5%) and a blend of CMC : SBR (1 : 1) (CMC: carboxymethylcellulose, DuPont; SBR: styrene–butadiene rubber, MTI Corp.) in water (VWR Chemicals). We mixed the binder with the active materials and the conductive carbon in a mixer (THINKY ARE-250) and cast the slurry on Al foil. The tapes were dried overnight under vacuum at a temperature of 100 °C for NMP-based ones, and 60 °C for the water-based ones. Then the electrodes were cut into 13 mm diameter disks with a steel puncher. The loading of HC and HC–Sn materials is 3–4 mg cm^−2^. For electrochemical testing, we prepared 2032-type coin cells in the half cell configuration in an argon-filled glove box (200 B, MBraun, Germany; H_2_O < 0.1 ppm, O_2_ < 0.1 ppm), with two layers of porous glass separators (Whatman GF/A) and Na metal as the counter electrode (prepared from a dry stick, Thermo Scientific, 250 μm thickness). We used 90 μL of electrolyte, and we tested 1 M NaPF_6_ in EC : PC (1 : 1) and in diglyme. Capacity retention tests were conducted at *C*/10 (1*C* = 250 mA g^−1^) and at the end of the discharge a CV step was added until the current reached a value of *C*/30. For the rate capability tests, we subsequently increased the *C*-rate from *C*/10 to 2*C* and increased the cutoff limit of the current in the CV step from *C*/30 to *C*/3. The cutoff voltage for all the tests was 0.002–2 V *vs.* Na^+^/Na.

## Results and discussion

### Structure of carbons and composites

**Fig. 1:**
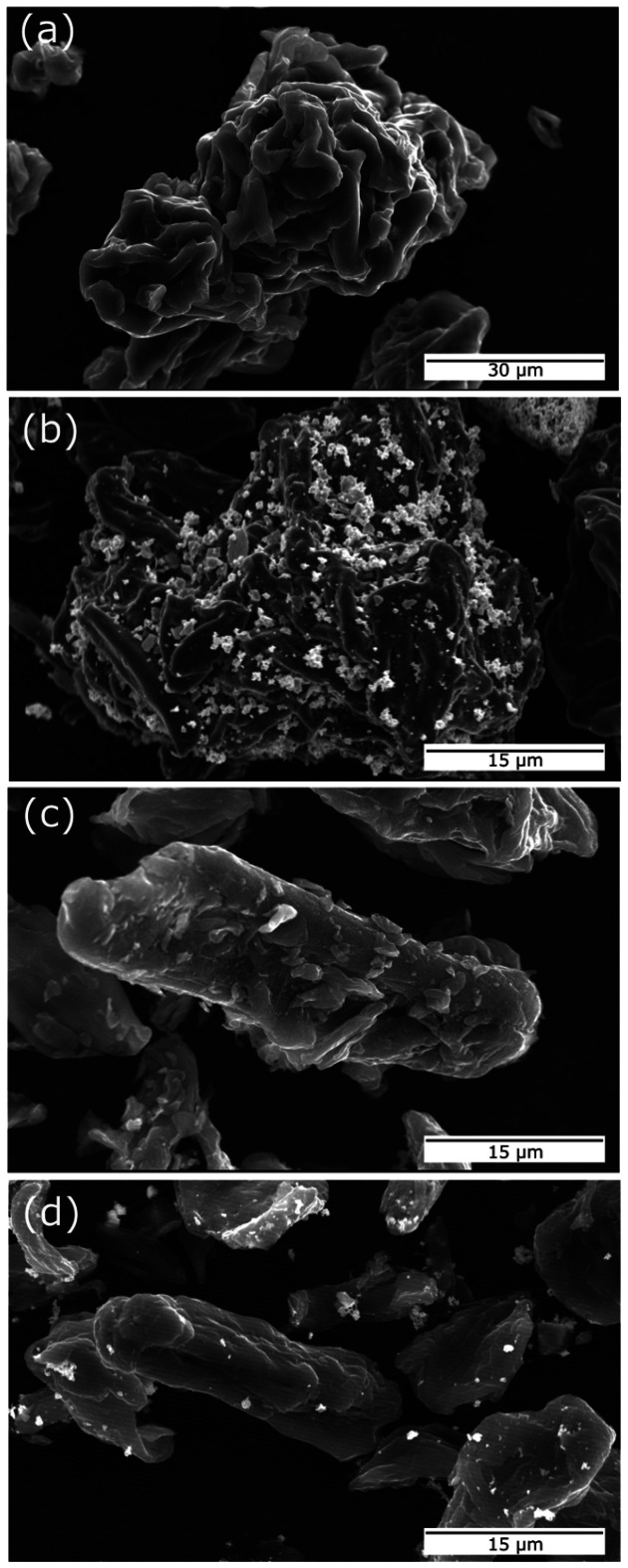
SEM images of (a) hard carbon from colloidal cellulose (CC), (b) composite CC-Sn, (c) hard carbon from fiber cellulose (FC), and (d) composite FC-Sn.

Hard carbon (HC) and HC–Sn composites were synthesized as described in the methods section. SEM images (Fig. 1) show the morphology of the samples made with different starting cellulose precursors. Clear differences between them can be observed: the HC obtained from colloidal cellulose named CC reveals a rough surface and a potato-like shape, while the HC obtained from fibrous cellulose named FC is composed of elongated and smoother particles. More SEM images are shown in Fig. S1 with lower magnification for significant statistical sampling. The Sn-containing composites are named CC-Sn and FC-Sn. During the spray-drying process, Sn nanoparticles find space in the cavities of CC, and they can be located on its surface. In the case of FC, Sn nanoparticles seem to have less surface roughness and appear to lay on the surface. Despite some agglomeration (visible in Fig. S1), Sn appears to retain its nanometric size and it is better accommodated on the surface of CC rather than on FC.

**Fig. 2:**
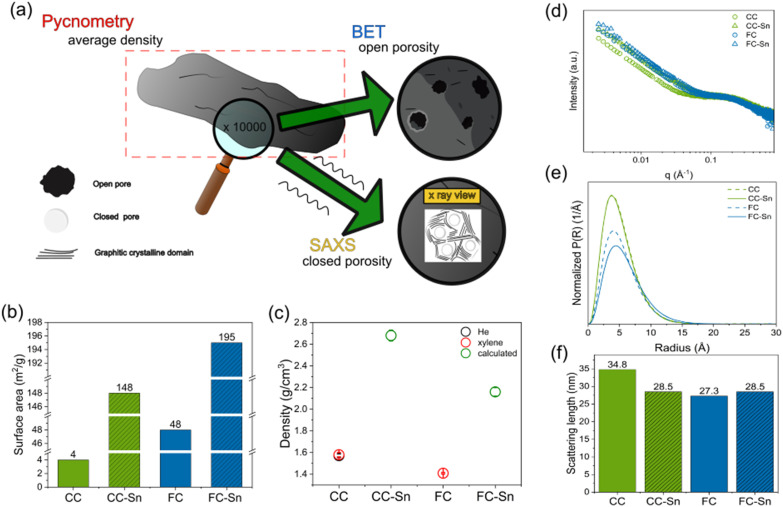
(a) Qualitative sketch of the structure of HCs. Pycnometry gives the average density of the entire particle, while suitable magnification of the surface (SEM, BET) shows open pores (meso/micropores). An X-ray view (SAXS) shows bulk closed micropores encapsulated in defective graphitic domains. (b) Surface area values of the samples obtained *via* BET. (c) Density values from pycnometry and (d) SAXS patterns of HCs and composites. Tin incorporation results in an increase of the HC density and a shift towards higher intensity in SAXS patterns. (e) Pore size distribution and (f) scattering lengths of HCs and composites.

Several methods have been used to highlight the differences between HCs and between HCs and the respective HC–Sn composites, as summarized in Fig. 2. Firstly, the surface area of the samples has been measured *via* the BET method. Fig. S2 displays the curves of the volume of adsorbed (desorbed) N_2_ as a function of the relative pressure. The effect of the spray-drying process on the HC surface area (without Sn addition) has been determined as well. Fig. 2b and Table S1 report the measured BET surface areas of all the samples. A quick comparison of the HCs reveals that CC exhibits a lower surface area than FC (due to CC's bigger particle size, see Fig. S1), highlighting the morphological differences between these two samples. In our study, the morphology of HC plays a big role, since usually in the literature different values of surface area are observed for samples treated at different temperatures (usually between 1000 and 1600 °C), while our HCs have been treated at the same carbonisation temperature of 1400 °C.^39,40^ So the difference in the precursors makes the surface area of FC larger than that of CC by one order of magnitude. As already well known in the literature, a low surface area is beneficial for sodium storage, as it determines a higher initial coulombic efficiency since less Na is irreversibly trapped in the SEI.^41,42^ For this reason, we expect different behaviour for the HCs during electrochemical cycling. Nevertheless the two hard carbons have different particle sizes, and we also expect this parameter to have a strong effect on the electrochemistry. The spray-drying process alone (without Sn) has a huge impact on HCs, since they both experience an increase in surface area probably due to the formation of surface defects during the process (Table S1). After spray-drying of HCs with Sn nanoparticles, the composites show a further increase in surface area, due to the presence of nanoparticles on the surface of HCs that make N_2_ adsorption more favourable. Moreover, Fig. S3 represents the pore size distribution of the samples and Table S2 their pore width, as obtained *via* DFT methods.^36^ Note that being determined by BET, these porosities refer to the accessible (open) porosity on the surface of the particles, as opposed to the internal (closed) porosity existing in HCs, which we will discuss later. One can observe that the pore width of the two HCs does not appear to be significantly different. On the other hand, both composites show bigger pore widths compared to the pristine HC; this provides evidence of Sn surface modification. In previous studies the effect of temperature on HC pore width has been taken into account, revealing that a higher synthesis temperature is translated into bigger and less abundant pores compared to the ones observed at lower synthesis temperatures.^39^ In our study the discrimination among our HCs is represented by the different cellulose precursors only, therefore a strong and clear correlation is not present. CC shows a variegated outer porosity, since micropores and mesopores are present in comparable amounts with multiple pore widths. The most abundant micropores have a width of ≈7 Å while the most abundant mesopores have a width of ≈55 Å. FC has more and smaller micropores than CC with a width of ≈6 Å, and a small amount of mesopores with a width of ≈60 Å. After the spray treatment without Sn, the micropores on the CC sample become predominant and more abundant than in CC, while in FC a multiplicity of micropores are formed with widths that cover the range between 5 and 10 Å. The spray-drying step is crucial in the morphological modification of the HC. After Sn mixing, the two composites exhibit similar features, with a comparable abundance of micropores.

Our aim is to increase the volumetric energy density of sodium anode materials, so we measured the density of the materials by glass pycnometry with xylene (Fig. 2c). The value of the density of the HCs lays in the expected range. Helium gas pycnometry was used as well, but only for CC we get a value comparable to the one from xylene pycnometer, which we believe to be trustworthy. The reason behind the mismeasured density for FC (that appears to be higher than graphite) could be ascribed to its surface area, that is one order of magnitude higher than CC. Since the pycnometer measures the difference in pressure between the analysed sample and a blank reference when a gas flows in the instrument to determine the volume of interest, if the probing gas is partly trapped (adsorbed) on the material we can expect a wrong density measurement. The situation does not improve when FC undergoes a degassing process at 300 °C under vacuum before the pycnometry measurement. Similarly to FC, the measured density for the composites is far from the expected values for both pycnometers. We can instead estimate the density of the composites is given by the sum of the densities of HC and Sn multiplied by the nominal weight % of each. From these values (determined in the next section) we obtain 2.68 ± 0.05 g/cm^3^ for CC-Sn and 2.16 ± 0.04 g/cm^3^ for FC-Sn. We believe these density values for the composites to be reliable, indicating that FC-Sn has a density close to graphite, while CC-Sn has a density well above.

Beyond the surface area of the samples, HCs are mainly known for their internal structure.^43,44^ The inner structure of the best performing HC is abundant in micropores, and in most cases they are closed pores.^45–47^ These features enable the HC to avoid parasitic reactions with the electrolyte, which cannot access the closed pores and contribute to the formation of a thin SEI. Only Na can access the closed pores during the electrochemical discharge step, leading to the huge plateau usually observed at ≈10 mV *vs*. Na^+^/Na, often attributed to Na plating in such closed pores. To probe these pores, we used small angle X-ray scattering (SAXS), as reported in Fig. 2d. In the SAXS patterns, a shift towards higher intensity is observed at low *q* values in the composite patterns, and this phenomenon can be attributed to the effect of the difference in electron density (*ρ*) between Sn and C. According to SAXS theory, the intensity follows

1 *I*(*q*) ∝ Δ*ρ*^2^

and in HC samples the only contribution comes from C and air present in the closed pores and surrounding the HC. In the composites, we add another contribution, coming from Sn particles and air. Since

2 *ρ*_Sn_ − *ρ*_air_ > *ρ*_C_ − *ρ*_air_

we justify the observed intensity shift. In the high *q* region, we observe the shoulder describing the closed micropores, and it is present in all our materials with a good overlap. We can state that no significant difference in the micropores is induced by Sn incorporation, as expected.

Based on previous works on *operando* SAXS on HC,^48^ we fit our data using different contributions:

3 *I*(*q*) = *I*_particles_(*q*) + *I*_pores_(*q*) + *I*_Sn_(*q*) + *I*_background_

where the particle term is a power law (Porod model) and the background term is a constant. For the pore contribution, a spherical particle model and Schulz–Zimm distributions are used, while for the Sn term we use the LogNorm distribution. SAXS fits are shown in Fig. S4, and the effect of the Sn contribution is shown in Fig. S5, where we subtract the *I*_particles_(*q*) term to show that the *I*_pores_(*q*) term alone cannot fit the experimental data properly. The pore size distribution is shown in Fig. 2e, and for all the materials the average pore radius falls in a range that is in good agreement with the value of HC synthesized at 1400 °C in the literature.^39,48^ However, we can find some differences in these values among our materials, since the samples CC and CC-Sn have an average pore radius of 3.93 Å, while FC and FC-Sn have values of 4.25 and 4.55 Å respectively, and fewer pores. Based on recent studies on the sodiation of HC, it is found that Na^+^ experiences a transformation to quasi-metallic behaviour and agglomerates in clusters in closed pores.^49–51^ Bigger closed pores can store more quasi-metallic Na ions, therefore a large size and high abundance are the key parameters for high Na^+^ storage in HC materials.^52^ These differences are supposed to give different performances in electrochemical tests, as we verify in the following.

We also extrapolate the values of the characteristic scattering length of the physical object (Fig. 2f), by fitting the region at low *q* with the power law only, and recording the *q* value when the fit shows an error of 10% (Fig. S6), and applying the following formula:4
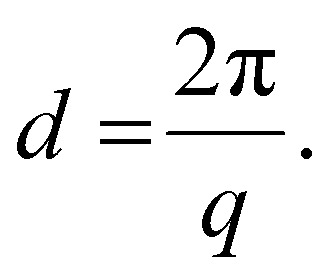


The power law describes the scattering behaviour of a sharp surface of a material in a medium.^53,54^ A deviation from this regime indicates that the interface is not well defined anymore, but it becomes more diffuse since the difference in electronic density of the two scattering media gets smaller. In the SAXS pattern this is translated into a more gradual transition with increasing *q*, since the intensity does not decay as fast as in the low *q* region anymore. The structure of HC is complex and rich in defects, therefore it is tough to give an absolute interpretation of this physical object. From the reported HC structural models,^8^ it can be interpreted as the size of aggregated primary particle clusters on top of secondary particles that determine the disruption of the evenness of the surface. This makes the scattering length an indicator of the roughness of the material's surface, since for an ideal smooth particle we expect the power law to describe a wider range of *q* compared to an irregular surface. A comparison of CC and FC shows that the power law has a bigger range of validity for FC than CC since it shows a higher *q* limit value, confirming that FC particles have a more regular shape and their surface is more even with fewer defects than CC. In the composites, CC-Sn experiences a decrease in scattering length compared to CC, indicating that Sn is located in the roughness of CC and makes its surface more even, while FC-Sn shows a slight increase in the scattering length, hinting that Sn increases the roughness of FC surface. This result is in agreement with SEM images of the materials, where the Sn distribution on the HCs is visible, suggesting that what we observe at the micrometre scale could also be reflected at the surface of the particles. A qualitative model of the HCs is shown in Fig. 2a, representing the open pores analysed *via* BET and the closed pores determined by SAXS.

In order to characterize the carbon matrix, we also performed Raman spectroscopy. The spectra of all the materials are shown in Figs. S7 and S8. In the spectrum of carbonaceous material, we can find the presence of the G band (*ca.* 1600 cm^−1^) due to the in-plane stretching of sp^2^ carbon atoms with *E*_2g_ symmetry, and the D band (1350 cm^−1^) due to the breathing of carbon atoms with *A*_1g_ symmetry.^55–57^ The D band is usually related to the defectiveness degree of the carbon material, and it is generated by the presence of carbon rings in the structure.^55^ The ratio between the intensity of the D band to that of the G band is a useful value to compare different carbonaceous materials, in order to evaluate the defectiveness degree of the structures.^58^ The spectra of the two hard carbons are reported in Fig. S7. We adopted the Sadezky model to deconvolute the spectra, so we find the G and D bands.^59^ In this model the D band can be deconvoluted into multiple contributions, so we have the peaks of crystalline graphite microdomains (D1) disposed in disordered domains (D3, D4) and some general amorphous carbon vibrational contributions.^60–62^ The parameters used for fitting are summarized in SI (Table S3). Here we report the first and second order of carbon, where the overtone bands (2D, 2D′) and the fundamental lattice vibration modes (D + G) can be found.^63^ The presence of Sn in the composites is revealed by the peaks at low Raman shift values. The Raman spectrum of Sn nanoparticles is used as a reference (Fig. S8f). In Fig. S8e we show the *I*_D_/*I*_G_ values of our materials. The two HCs show similar values of ordering, so their morphology does not have a significant impact on the defectiveness that can be probed with Raman spectroscopy. However, in the composites we can appreciate a small change in this value. This suggests that the spray-drying process induces or increases a mode in the D band, resulting in an increasing defectiveness degree of the material, perhaps near the surface.

In summary, the results for the characterization of the HC and HC–Sn materials revealed that the mesostructure (surface area, scattering length and defectiveness) was strongly dependent on the morphology of the HC, and it was affected by Sn incorporation *via* the spray-drying process. Comparing the HC samples, we find that CC shows a surface area one order of magnitude smaller than that of FC, probably due to its bigger particles and higher roughness, as confirmed by SEM, BET and SAXS measurements. On the other hand, FC shows a lower density and bigger closed pore sizes compared to CC. With both carbons, when Sn is incorporated, we observe an increase in surface area and *I*_D_/*I*_G_; this is a sign that Sn nanoparticles influence the active sites on the surfaces available for gas adsorption and induce the activation or enhancement of defective modes detected by Raman spectroscopy. On the other hand, Sn incorporation results in a decrease of roughness in CC, which is a sign that the nanoparticles find room in hard carbon cavities, while it results in an increase of the roughness of FC, where no cavities are present, as revealed by SAXS and SEM. The microstructure (inner pore abundance and size) is instead not modified by Sn incorporation, which is indeed more affected by other parameters such as the synthesis temperature.^40,63^

### Determination of Sn content

A potential drawback of spray drying is the unknown Sn loss during the process. Even though we prepared a Sn nanoparticle suspension for spray drying targeting 30% Sn content, it must be verified what amount is effectively incorporated. We then propose different methods to estimate the Sn amount in our materials by using scattering, thermal analysis, imaging and electrochemistry.

XRD is a valid tool to estimate the amorphous content of a material, by collecting the pattern of a blend of the sample with a known amount of a crystalline standard.^64^ Here, we prepared one sample of our composites and Si as a reference in a ratio of 10 : 1, packing a capillary to conduct the measurement. In Table 1 we report the results of the amorphous phase estimation in the composites. In Figs. S9 and S10 the patterns of CC-Sn and FC-Sn are reported with the respective Rietveld refinements. By this method we find an amount of the crystalline phase that is around 18.4% for CC-Sn and 11.4% for FC-Sn. We also tried to employ LaB_6_ as a reference material (Table S4), but we obtained an estimated crystalline phase content of around 5% for CC-Sn and 5.5% for FC-Sn. These low values are due to the huge difference in density between our materials and LaB_6_, which does not guarantee the formation of a homogeneous blend of sample and reference, so the measurement is strongly affected by sample preparation issues. Therefore we deem only the measurements performed with Si trustworthy.

**Table 1:** Data of XRD measurements and refinements with Si to quantify the amorphous phase in the composites

Material	Reference	*λ* (Å)	AM : reference	Amorphous content (%)	*R*_Bragg_ reference (%)	*R*_Bragg_ Sn (%)
CC-Sn	Si	0.70932	10 : 1	81.63 ± 1.69	6.99	6.94
FC-Sn	Si	0.70932	10 : 1	88.64 ± 1.05	6.56	4.66

We also find that during the synthesis in water, some Sn undergoes oxidation resulting in poorly crystalline SnO and SnO_2_, which can be observed by the broad peaks shown in the diffraction patterns in Figs. S9 and S10. Due to the uncertainty introduced by these side phases, other methods to determine the Sn content were also investigated.

**Fig. 3:**
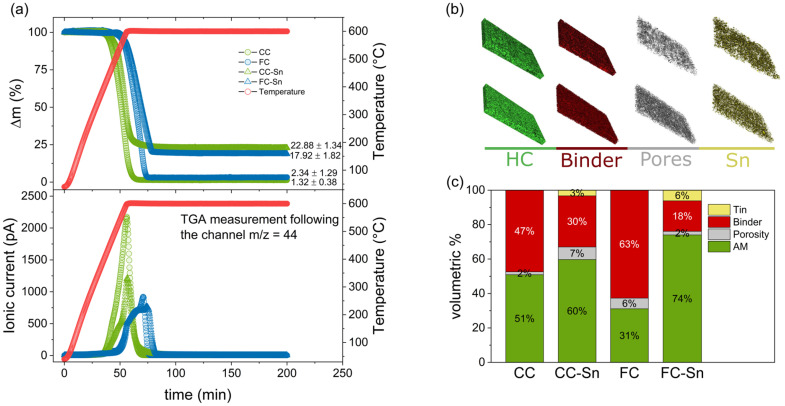
(a) Residual mass of the HCs and composites after TGA-MS in air and relative ionic current given by CO_2_ evolution. (b) 3D XRM reconstructions of the section of composite electrodes (CC-Sn on top and FC-Sn on the bottom). (c) Volumetric% of electrode components.

A technique that does not require assumptions about the crystal structure of the materials is thermogravimetric analysis (TGA), and it has already been used in the literature to determine the Sn amount in Sn composites used as anodes for lithium-ion batteries.^65^ In Fig. 3a we report the mass loss as a function of time during the heating process up to 600 °C in air. Fig. S11 shows the TGA experiment of Sn nanoparticles alone. In the end, the residual mass (Δ*m*_Sn_) recorded for Sn was 117.05 ± 0.28%. This value lies between the ideal values obtained for the formation of SnO (113.48%) and SnO_2_ (126.95%), hence the final chemical formula of the compound we obtain after Sn heating is SnO_*x*_ with 1 < *x* < 2. Knowing the residual masses of HC (Δ*m*_HC_) and composites (Δ*m*_HC–Sn_) and assuming that Δ*m*_HC–Sn_ is composed of the sum of Δ*m*_HC_ and Δ*m*_Sn_ we can use the following equation to determine the Sn amount in our materials:5
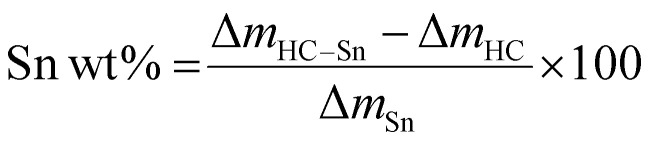


By this method the amount of Sn in CC-Sn is found to be 18.42 ± 1.19%, and in FC-Sn it is 13.31 ± 1.90%. These values still confirm that some Sn is lost during spray drying, and we believe they are trustworthy as they do not depend on any features of the samples. Moreover, Fig. 3a also exhibits a mass spectrometer analysis (TGA-MS), where we follow the evolution of CO_2_ during the heating process (*m*/*z* = 44 channel) confirming that all the mass loss comes from the oxidation of carbon. Direct proof of the effect of Sn in HC materials is definitely the increase in gravimetric capacity (mAh g^−1^). As noted previously, in sodium ion batteries Sn shows its theoretical capacity and good performances in terms of capacity retention and electrode stability in ether-based electrolytes. Although we decided to focus on conventional carbonate-based electrolytes for this study, the use of ethers can help us reveal the Sn content in our composites. In Fig. S12 the electrochemical performances of CC-Sn and FC-Sn with 1 M NaPF_6_ in diglyme are shown. Assuming that the total capacity of the composites is given by the HC and Sn capacities multiplied by their weight %, we obtain6
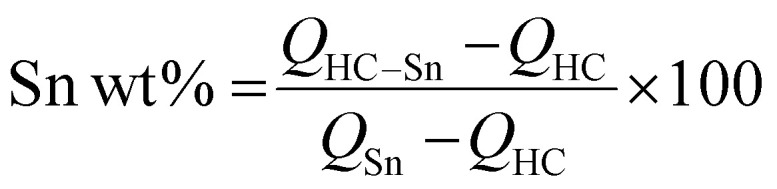
where *Q*_HC–Sn_ is the experimental capacity of the composite, *Q*_HC_ is the experimental capacity of the HC and *Q*_Sn_ is the theoretical capacity of Sn (847 mAh g^−1^). From this equation we obtain a Sn amount for CC-Sn of 20.87 ± 0.18%, and 13.65 ± 0.17% for FC-Sn (based on the capacity of the 10^th^ cycle). These values are in very good accordance with TGA results.

Finally, we employed X-ray tomographic imaging to further confirm the composite structures and Sn distributions. Here, a known volume of the samples cast on aluminium (electrodes) was cut and measured with X-ray microtomography (XRM). Starting from the known percentage of the components of the electrodes, it is possible to calculate the percentages of active material, binder, and pores. The active material can moreover be distinguished between HC and Sn, so then a volumetric percentage of the components is given. In Fig. 3b we report a 3D reconstruction of the composite electrodes and in Fig. 3c the results of the data treatment to define the volumetric percentage of each component in the electrodes. The 3D reconstruction of the HC electrodes can be found in Fig. S13. The weight percentage (wt%) of each component was calculated based on the volumetric ratios extracted from the XRM data, with two key considerations: porosity was excluded from the weight percentage calculations due to the lack of density information, and the density of the binder plus conductive component was assumed to be 1.5 g cm^−3^. For the HC–Sn composites, the density values obtained by pycnometry were used. These assumptions ensured consistency in the estimation of electrode composition.

We used the equation7
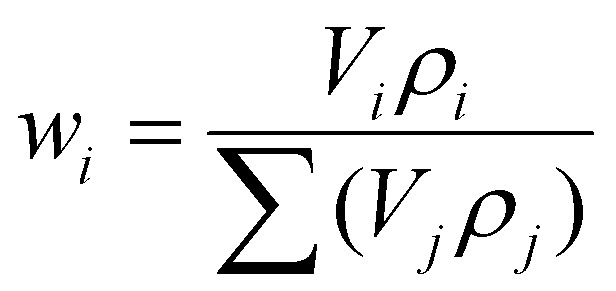
where *w*_*i*_, *V*_*i*_, and *ρ*_*i*_ are, respectively, the weight fraction (%), volume fraction (%) and density (g cm^−3^) of component *i*, and *j* indicates all the components (HC, Sn, binder plus conductive carbon). The Sn weight % results to be 14.80% for CC-Sn and 19.33% for FC-Sn.

**Fig. 4:**
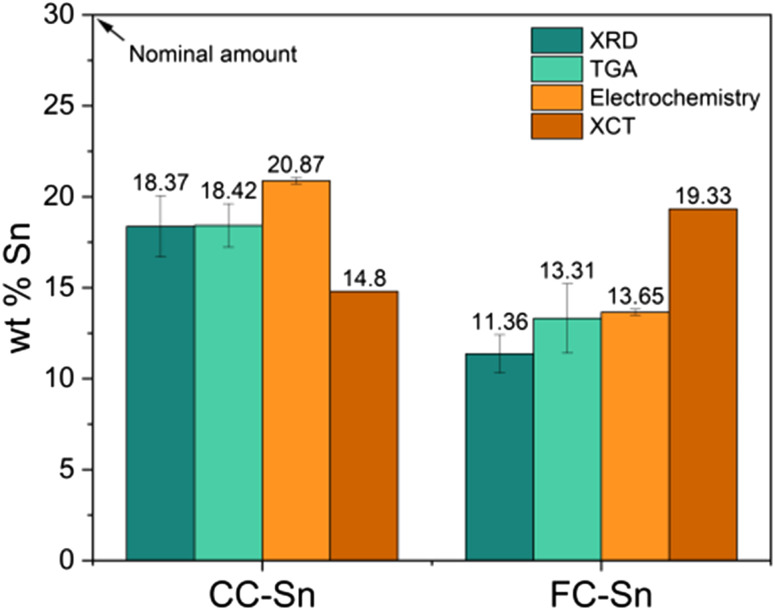
Weight % of Sn in the CC-Sn and FC-Sn composites determined with different techniques: XRD with Si reference (dark aquamarine), TGA-MS (pale aquamarine), electrochemistry with diglyme electrolyte (orange) and X-ray microtomography (brown).

Finally, all the results obtained from different techniques are summarized in Fig. 4. We can conclude that CC-Sn composites feature a higher Sn content, of the order of 18–20%, while FC-Sn has a lower content, approximately 13–15%. As TGA estimation appeared to be reliable, it was also performed on composites containing a higher amount of Sn, where a 1 : 1 HC : Sn weight ratio was targeted (labelled CC-Sn_50 and FC-Sn_50). The composites revealed Sn amounts of 26.87 ± 2.26 wt% and 23.89 ± 1.12 wt%, respectively. This result indicates that for spray drying on the laboratory scale the reactor size allows us to get a reaction yield (Sn content in composites *vs.* Sn content targeted) of approximately 50%. Moreover, the morphology of CC enables a better incorporation of Sn than FC, since we constantly record a higher Sn amount for this hard carbon. As is often the case, it can be expected that industrial spray-dryers would achieve better yields due to their larger size and reduced surface area.

### Electrochemical results

**Fig. 5:**
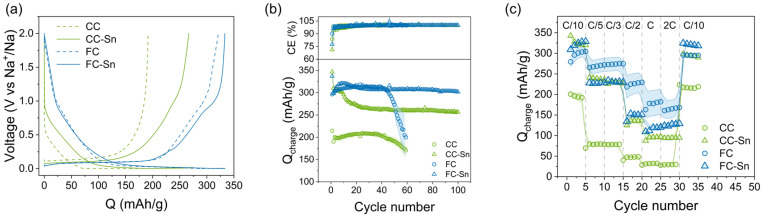
(a) Voltage profiles (10^th^ cycle), (b) capacity retention (at *C*/10 rate) and CE and (c) rate capability tests of the samples. The composites show higher gravimetric capacity and better stability than the relative HC with increasing number of cycles. Testing is performed on half cells at 25 °C over the voltage window 0.002–2 V *vs.* Na^+^/Na.

Although it has been shown that the best electrolyte choices for metal anode electrodes are ether-based, for reasons of sustainability, cost, and adaptability to established Li-ion technology, we carried out electrochemical tests using conventional carbonate-based electrolytes. All our samples were tested in half cells *vs*. sodium metal with 1 M NaPF_6_ in EC : PC as the electrolyte, and the electrochemical results are reported in Fig. 5.

It has been shown that CMC and CMC : SBR binders perform better for HC materials than the conventional PVdF binder.^38^ For this reason we firstly prepared electrodes of hard carbons with CMC : SBR binder using an AM : binder ratio equal to 95 : 5. We confirmed the successful usage of CMC : SBR for our FC electrode, while for CC the use of CMC : SBR was problematic and led to poor electrochemical performances (Fig. S14). To understand the origin of this phenomenon, we compared CC electrodes prepared with CMC : SBR and with PVdF by SEM. The SEM images are reported in Fig. S15, and since we have used the same parameters for both, a comparison can be reasonably made. The effect of CMC : SBR binder (Fig. S15a) is to form a uniform thick (and likely passivating) layer on the surface of the electrode, while the surface of the electrode prepared with PVdF (Fig. S15b) looks thinner and accessible, and the CC particles are simply bound together. We then tried to optimize the amount of CMC : SBR in a CC electrode and identified the 96.5 : 3.5 formulation as enabling contact between the particles but not the formation of a passivating layer. However, the galvanostatic cycling test shows unstable behaviour of the CC electrode, with a coulombic efficiency that overcomes the 100% value in several cycles (Fig. S14). On the other hand, the electrode formulation with PVdF shows stable capacity behaviour even if providing a poorer performance. For this reason we choose to use the conventional PVdF binder for the CC and CC-Sn couple, preferring a stable electrode to better understand the effect of Sn in the composite material. Future work will be dedicated to the stabilization of CC with CMC : SBR binder and then transferred to the composite formulation. In Fig. 5a we show the voltage profile after the initial formation (10^th^ cycle) of all our materials using PVdF binder in the electrode formulation for CC and CC-Sn and CMC : SBR for the FC and FC-Sn couple. In both cases, the contribution of Sn can be clearly seen in the shoulder present at 0.75 V *vs*. Na^+^/Na during the charging step.^28,66^ The difference in capacity among the pristine HCs is also reflected in the composites and can be attributed to the size differences of closed pores in the materials and the roughness of the surfaces. FC and FC-Sn show larger closed pores than CC-Sn and CC, thus the higher capacity of the plateau region at low voltage values. In Fig. 5b the capacity retention of the samples is reported. The improved capacity of CC-Sn compared to CC results in 55 mAh g^−1^ being gained in the 30^th^ cycle, and moreover the stability of CC is highly surpassed. Regarding the FC-Sn composite, despite a small improvement of the initial capacity (20 mAh g^−1^ more than FC), both samples perform similarly after 10 cycles. However the stability of FC has been clearly improved after Sn incorporation; this is visible after 50 cycles. In Fig. 5c the rate capability test of our samples is shown. CC-Sn is superior to CC in every step, as a result of higher capacity, better stability and faster kinetics. For FC-Sn the situation is different, since it provides more capacity than FC, but it appears to suffer from poor kinetics; FC indeed shows better kinetics at higher *C*-rates. However, with smaller error bars, FC-Sn samples show more reproducible electrochemical behaviour. Overall, these rate capability tests provide a first indication but they need to be repeated in the 3 electrode cell configuration, which will be the subject of future work.

Considering the calculated density values (Fig. 2c) in combination with the techniques in Fig. 4, our composites provide a higher volumetric capacity than HCs as well, resulting after 50 cycles in 697.30 mAh cm^−3^ for CC-Sn and 662.98 mAh cm^−3^ for FC-Sn (in comparison with 301.83 mAh cm^−3^ for CC and 384.53 mAh cm^−3^ for FC). These results confirm that our approach leads to anode materials with increased volumetric energy density, considering that in LIBs a graphite-based anode provides 814 mAh cm^−3^ (assuming a specific gravimetric capacity of 370 mAh g^−1^ and a density of 2.20 g cm^−3^). Furthermore, we investigated the possibility of increasing the active material percentage in the electrode, testing the formulations of AM : conductive carbon : binder equal to 85 : 10 : 5 and 90 : 5 : 5. The capacity retention tests are reported in Fig. S16, clearly exhibiting worse performances in both cases with respect to the formulation 70 : 20 : 10. This demonstrates the importance of the conductive carbon additive when Sn is present in the electrodes. Indeed some of the Na–Sn alloys that form during electrochemical discharging (*e.g.* NaSn) may have a high melting point and low electrical conductivity,^67^ and do not guarantee the optimal working of the electrode. Once it was found that the conductive carbon was fundamental for the electrochemistry of the composites, we tested samples with a higher Sn content, CC-Sn_50 and FC-Sn_50. The results of electrochemical tests are shown in Fig. S17, and they reveal different behaviour for the two composites. Initially, both show higher capacities than those of CC-Sn and FC-Sn, as expected. After a few cycles, however, FC-Sn_50 suffers from the higher Sn amount, resulting in a lower capacity than that of FC-Sn, likely due to Sn agglomeration and subsequent pulverization during cycling. On the other hand, surprisingly CC-Sn_50 shows a higher capacity than that of CC-Sn, confirming that in this material the activation of Sn is more pronounced in terms of capacity gained through the morphology of CC in which Sn particles are embedded. This indicates that to increase the Sn content a suitable HC morphology is paramount. It should be noted, however, that the performances of CC-Sn_50 after 10 cycles are inferior to those of FC-Sn, indicating that for stable cycling at values close to 300 mAh g^−1^ large amounts of Sn are not required.

Finally we tested the effect of the spray-drying process (without Sn) on HC electrochemistry, as shown in Fig. S18. The water-based spray-drying process clearly induces a degradation in performance, as both the gravimetric capacity and the initial coulombic efficiency (ICE) are lower than those of pristine HC. This behaviour is likely related to the increase in surface area observed during the spray-drying process,^41^ as shown by BET measurements. The presence of Sn assumes an important role in the CC-Sn and FC-Sn composites: even though we observe a decrease in ICE from 83.7% to 71.2% for the CC/CC-Sn couple and from 89.9% to 77.5% in the FC/FC-Sn couple, the subsequent cycling stability is significantly higher when Sn is present. Moreover, the difficult cell balancing due to lower ICE can be eventually overcome by alternative strategies like anode presodiation or addition of sacrificial salt.

### Sodiation mechanism determined by *operando* XRD and *ex situ* SEM/EDX

**Fig. 6:**
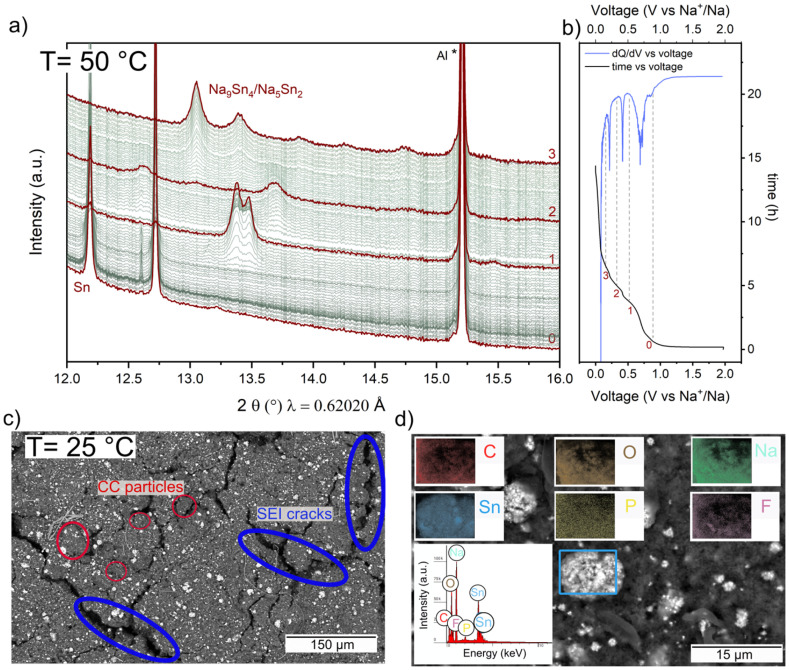
*Operando* SXRPD of CC-Sn at 50 °C and *C*/10 (a) and the corresponding voltage profile and d*Q*/d*V* curve (b). We highlight the patterns corresponding to observed intermediate crystalline phases. The peak marked with * corresponds to Al from the current collector. (c) *Ex situ* SEM and EDX (d) of CC-Sn after a full discharge at 25 °C.

Synchrotron X-ray powder diffraction (SXRPD) is one of the most powerful techniques for the study of complex phenomena that occur in battery processes.^68^ Following the transformation of the electrode materials during coulometric titration gives fundamental information regarding structural changes and phase evolution. Sn, as a negative electrode material, has been investigated in the literature *via operando* XRD, and usually not all the alloys suggested by the Na–Sn binary phase diagram are observed to form.^18,69^ One would expect to observe the evolution of Sn, NaSn_5_, NaSn, Na_9_Sn_4_ and Na_15_Sn_4_ phases, but actually only the high sodium content phases are observed during electrochemical discharge (*i.e.* typically Na_9_Sn_4_ and Na_15_Sn_4_).^33,69,70^ It is suggested that the remaining phases are kinetically hard to crystallize. More details are presented in the study by Stratford *et al.*,^71^ where some other structures are proposed as intermediate Na–Sn phases, based on NMR and PDF data. The same challenge was reported by Palaniselvam *et al.*,^72^ where the *operando* XRD of Sn was investigated for tin–carbon composite electrodes. Here, the authors confirmed the lack of some expected phases by showing the mismatching of the *in situ* patterns with the reference ones. Since we are interested in confirming the activity of Sn in our composites as well as in verifying the results reported in the literature, we decided to carry out a synchrotron X-ray powder diffraction (SXRPD) measurement of the CC-Sn composite, which had a higher Sn content. In Fig. 6a we show the *operando* SXRPD of CC-Sn at 50 °C during electrochemical discharge at *C*/10, with the voltage profile and the d*Q*/d*V* curve in Fig. 6b. A higher temperature was selected to enhance the slow kinetics of Sn phase formation.

At OCV, we can clearly identify the peaks of Sn. At the beginning of electrochemical discharging we observe a first voltage plateau at 0.7 V. Below 0.5 V the formation of Na–Sn alloys becomes visible. The d*Q*/d*V* plot (Fig. 6b) allows us to identify the processes encountered in the discharge curve, as we can match its peaks with the plateau in the voltage profile, and the minima with the stabilized intermediate phases. The reactions start with the initial phase marked by “0” corresponding to Sn metal and leading to the first effective plateau, at the end of which the first Na_*x*_Sn (0 < *x* < 1) intermediate phase forms, as marked by “1”; this was revealed by the disappearance of the Sn metal peaks in the SXRPD pattern, and the formation of a peak doublet around 13.2–13.5°. Based on the electrochemistry result, this phase corresponds to Na_1.08_Sn. The next plateau leads to the formation of a second unidentified phase “2” characterized by a clear reflection at 13.7° and nominally corresponding to Na_1.80_Sn. Presently we cannot attribute these intermediate phases to any known structure and the confirmation of the stoichiometry suggested by the electrochemistry will be subject of future work. Finally, the last plateau generates a final crystalline phase with a main reflection at 13° (marked by “3”). Once reaching 0.002 V we get a capacity of 563.45 mAh g^−1^, which can be divided into the contributions of CC and Sn. Since we expect Sn to contribute roughly 19% of the total capacity, we assume it provides 107.06 mAh g^−1^, which is the equivalent of the capacity provided by an electrode containing 19% of Na_4.99_Sn_2_. Our *operando* pattern at the end of discharging matches well with the Na_5_Sn_2_ phase, as also seen in Fig. S19. This crystalline phase has been observed in *operando* studies on Sn foil and film^73,74^ and powder electrodes^71^ in carbonate electrolytes, while in studies that used ether electrolytes it is mostly replaced by the Na_9_Sn_4_ phase. It has been shown that both crystalline phases Na_5_Sn_2_ and Na_9_Sn_4_ electrochemically crystallize in the space group *R*3̄*m*, therefore a solid solution with the Na_5−*x*_Sn_2_ composition is formed, starting from Na_9_Sn_4_, which gradually transforms into Na_5_Sn_2_ with the increase in occupancy of one Na site.^71,75^ Therefore it is impossible to unequivocally identify the final phase observed in Fig. 6, but the electrochemical results indicate it is close to the Na_5_Sn_2_ phase. To further confirm this, we conducted *ex situ* SEM-EDX measurements on a composite anode that was fully discharged in a half cell at 25 °C. Fig. 6c shows the SEM image of the CC-Sn electrode, and with the backscattering detector it is easy to identify the shiny particles that correspond to the Na–Sn alloys (see Fig. S20c for the FC-Sn electrode as well). Indeed, when the SED detector is used, no clear difference between the HC and Na–Sn alloy particles is present (Fig. S20a and S20b). After electrochemical discharging, the SEI has been formed and the expansion of Sn, once it has reacted with Na, is translated into the formation of some cracks on the surface of the electrode. In Fig. 6d we report the EDX analysis of the same electrode, with a focus on the shiny particles, in an attempt to quantify their elemental composition. As we expect, EDX reveals the presence of C, Sn, Na and O, with some traces of P and F coming from the electrolyte. All these elements are uniformly distributed on the target particle, and we were able to fit the EDX spectrum for elemental quantification. Based on the atomic weight of the elements of interest and considering that our measurement is not provided with a standard for accurate EDX elemental quantification, we expect a bigger error in the estimation of Na amount than for Sn, so we have performed the analysis on different areas of the electrode for a more reliable statistic. Repeating the measurement on 3 particles, *ex situ* analysis confirms a wt% ratio of Na/Sn equal to 2.4 ± 0.2 for CC-Sn and 3.1 ± 0.4 for FC-Sn, and these results confirm the non-complete sodiation of Sn, since the Na_3.75_Sn phase (Na_15_Sn_4_) has not formed. There is incomplete sodiation of tin in this work, so the absence of the high-sodium-content phase Na_15_Sn_4_ could be ascribed to the presence of HC, which shows its most significant contribution in the long plateau at low voltage values. The fact that the HC capacity observed in this *operando* experiment is higher than the values observed in half cells is most likely due to stronger electrolyte degradation at 50 °C at the low voltage of the HC plateau. Limited Sn sodiation during electrochemical discharging (occurring primarily at a higher voltage than the HC plateau) offers a recognizable advantage in the system as we experience an increase in the gravimetric capacity of the composite without incurring in huge electrode volume changes, which are expected to be confined to 247% compared to 388% reported for the Na_15_Sn_4_ phase,^20^ thus explaining the noticeable stability of our composites.

### Comparison of spray-drying with other methods of Sn incorporation

**Fig. 7:**
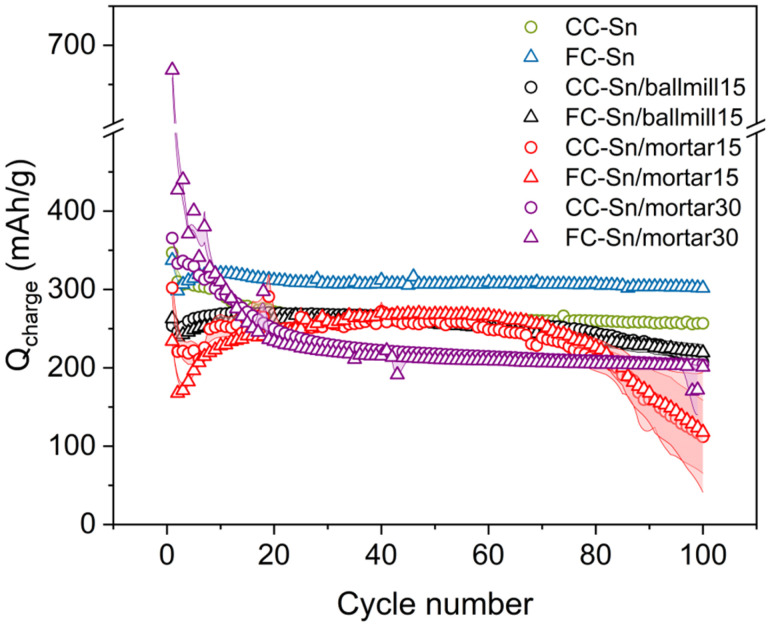
Electrochemical performance of spray dried samples *vs*. composites made by other methods (hand ground and ball milled). Here we report samples prepared with 30 and 15 Sn wt% in a mortar and the samples prepared with 15 Sn wt% in ball milling jars.

In order to prove the superiority of spray-drying synthesis over conventional routes, we also synthesized additional composites starting from the same chemicals. First of all, we tried to simply mix HC with Sn nanoparticles in a mortar, and we hand-mixed the blend for 10 min. We prepared two couples of batches, where the Sn amount was varied. For the first batch we used 30 wt% of Sn, as we did for the spray drying, and for the second batch we used 15 wt% Sn, as it was a closer value to the amount of Sn we effectively obtained in our spray-drying composites (Fig. 4). We labelled the samples CC-Sn/mortar30 and FC-Sn/mortar30 for the samples with 30 Sn wt%, and CC-Sn/mortar15 and FC-Sn/mortar15 when the Sn wt% was 15. Furthermore, we synthesized another batch *via* mechanical ball milling, as it is commonly used to prepare composite anode materials.^28^ Ball milling guarantees an energetic mixing of the precursor, offering a good comparison in our study; we used 15 wt% Sn for this preparation. We labelled these last samples as CC-Sn/ballmill15 and FC-Sn/ballmill15. We confirmed the expected Sn amount in all the new samples with TGA (Fig. S21). This time we know exactly the amount of Sn that we expect to be integrated into the samples, since the loss of material is negligible compared to spray drying (especially for the hand-mixed samples). This further highlights the feasibility of TGA as a technique to determine the unknown amount of Sn in composite materials. We tested the new materials electrochemically in the same configuration as the spray-dried samples, and their electrochemical performance is shown in Fig. S22. In the first cycle, the high Sn content samples (CC-Sn/mortar30 and FC-Sn/mortar30) show the highest capacity due to the Sn contribution to the sodiation process, but then they show rapid capacity fading (Fig. S22c). This can be explained by the huge volume expansion of Sn during the sodiation process, which results in continuous SEI destruction cycle by cycle and in cell failure.^15,26^ The low Sn amount samples prepared in the mortar show a poorer initial capacity compared to the high Sn amount ones, as expected (Fig. S22a). Moreover, insufficient HC and Sn mixing does not result in any improvement of capacity but in discontinuous and poorly reproducible behaviour that leads to capacity fading, once the 50^th^ cycle has been reached (as for pristine HCs). Regarding the ball-milled samples (Fig. S22b), in the sample CC-Sn/ballmill15 we record a capacity comparable to that of CC-Sn in the 20^th^ cycle, but there is a capacity drop afterwards. For the FC-Sn/ballmill15 sample we even record a lower capacity than those of the HCs. This feature can be explained by considering the pulverization of HC induced by hard ball milling, which not only decreases the particle size but also modifies the internal structure made of micropores.^52^ The low Sn content is then not sufficient to compensate for this lack of capacity. The electrochemical results for all composites are gathered in Fig. 7, which clearly demonstrate how HC–Sn composites made by spray drying may be a promising alternative to provide stable cycling above 300 mAh g^−1^, and with improved volumetric performances as well.

## Conclusion

In our work we synthesized HC–Sn composites by spray drying. Their physical properties were accurately tested by a combination of Raman spectroscopy, SAXS, SEM and BET analysis, and their electrochemical performances were tested in Na half cells. We faced the problem of determining the Sn content in our composites, and we successfully benchmarked a range of different techniques, showing that TGA, electrochemical measurements in diglyme electrolytes and XRD with a proper reference material gave comparable and trustworthy results. Based on this, we found that our samples contained on average close to 15 or 25 wt% Sn, depending on the initial Sn amount. The structural and morphological effects of spray-drying synthesis were determined, taking into account the amorphization of Sn nanoparticles and modification of the surface area of HC materials. We confirm that these parameters and mesostructure modification after Sn incorporation are the main reasons for the improvement in capacity with respect to HC, since the nanostructure of HC analysed with SAXS and Raman spectroscopy shows minor differences after spraying. The electrochemical effect of Sn was determined *via operando* synchrotron XRD, revealing the formation of two intermediate phases before the solid solution of Na_5−*x*_Sn_2_ was finally obtained; this increased the hard carbon capacity without incurring a dramatic volume expansion typical of high-sodium-content Na–Sn alloys. These results were also confirmed by *ex situ* SEM and EDX analysis of electrodes recovered at the end of electrochemical discharging. Moreover we verified the importance of an optimal electrode formulation, highlighting that a small difference in binder percentage could affect the electrochemistry performance of the materials. Different Sn contents in the composites were tested, revealing that a higher Sn content only gave an improvement in the electrochemical performances for appropriate HC morphologies, hence we reported an improvement for CC-Sn_50 on CC-Sn and a deterioration for FC-Sn_50 on FC-Sn. We also compared the electrochemical performance of our spray-dried samples with other reference materials, synthesized with the same reactants but using hand mixing and ball milling. Overall, our spray-dried materials showed the best capacity retention, since we performed an effective mixing of the precursors without modifying the internal structure of the HC. As a result, the HC matrix successfully helps to mitigate expansion and pulverization of the Sn nanoparticles, while retaining its sodium storage capacity. The increase in effective surface area after spraying also contributes to the improved performances. In this context, the morphology of HC plays a fundamental role, as in CC-Sn we observe a more noticeable increase in gravimetric capacity compared to CC, due to the favourable presence of roughness on the CC surface that allows for Sn storage, as confirmed by the SAXS scattering length value and SEM analysis. These features result in a volumetric capacity that approaches the one of graphite in LIBs. While for FC-Sn, which shows less surface roughness, we observe a smaller difference in electrochemical performance compared to that of FC. Nonetheless, FC-Sn is the best composite in terms of gravimetric capacity and especially capacity retention, which is consistently above 300 mAh g^−1^ after 100 cycles. In any case, it should be noted that due to the increased density, for both composite negative electrodes we observe an increase in volumetric capacity (and energy) as well. This represents a potential solution to a fundamental problem of commercial Na-ion batteries, making spray-drying synthesis a valid alternative to other methods to increase the gravimetric and especially volumetric performances of negative electrode materials.

## Conflicts of interest

There are no conflicts to declare.

## Supplementary Material

EB-001-D5EB00188A-s001

## Data Availability

Data for this article, including BET, SAXS, electrochemical testing, *operando* XRD, Raman are available at Zenodo at DOI: 10.5281/zenodo.15473104. Supplementary information (SI) is available. The Supplementary Information file includes additional low magnification SEM of the materials and electrodes, and SEM-EDX analysis of FC-Sn. Information extracted out of BET data are shown. Deconvolution of SAXS patterns and Raman spectra are plotted. Additional XRD, TGA and electrochemistry plots with glymes for Sn content determination are shown. Further electrochemistry data are: HC materials spray-dried without Sn, composites prepared by hand grinding and ball milling, composites by spray-dry with higher Sn content. See DOI: https://doi.org/10.1039/d5eb00188a.
