# Supplementary material for: Spray-dried hard carbon–Sn composites for energy-dense Na-ion batteries
Source: EES Batter. 2025 Oct 10;1(6):1596–611. doi: 10.1039/d5eb00188a (PMC12541904; doi:10.1039/d5eb00188a)
Supplement: EB-001-D5EB00188A-s001 [file EB-001-D5EB00188A-s001.pdf]

Supplementary Information (SI) for EES Batteries.
This journal is © The Royal Society of Chemistry 2025

SUPPORTING INFORMATION

Spray-dried Hard Carbon – Sn composites for energy-dense Na-ion batteries

Giovanni Gammaitoni[1,2], Gihoon Cha[3], Rajkumar Reddy Kolan[3], Silke Christiansen[3], François Fauth[4], Matteo Bianchini[1,2,*]

1 Faculty of Biology, Chemistry and Earth Sciences, University of Bayreuth, Universitätstraße 30, 95447 Bayreuth, Germany

2 Bavarian Center for Battery Technology (BayBatt), Weiherstraße 26, 95448 Bayreuth, Germany

3 Fraunhofer Institute for Ceramic Technologies and Systems IKTS, Äußere Nürnberger Straße 62, 91301 Forchheim, Germany

4 CELLS-ALBA Synchrotron, Cerdanyola del Vallès, 08290 Barcelona, Spain

[*] matteo.bianchini@uni-bayreuth.de

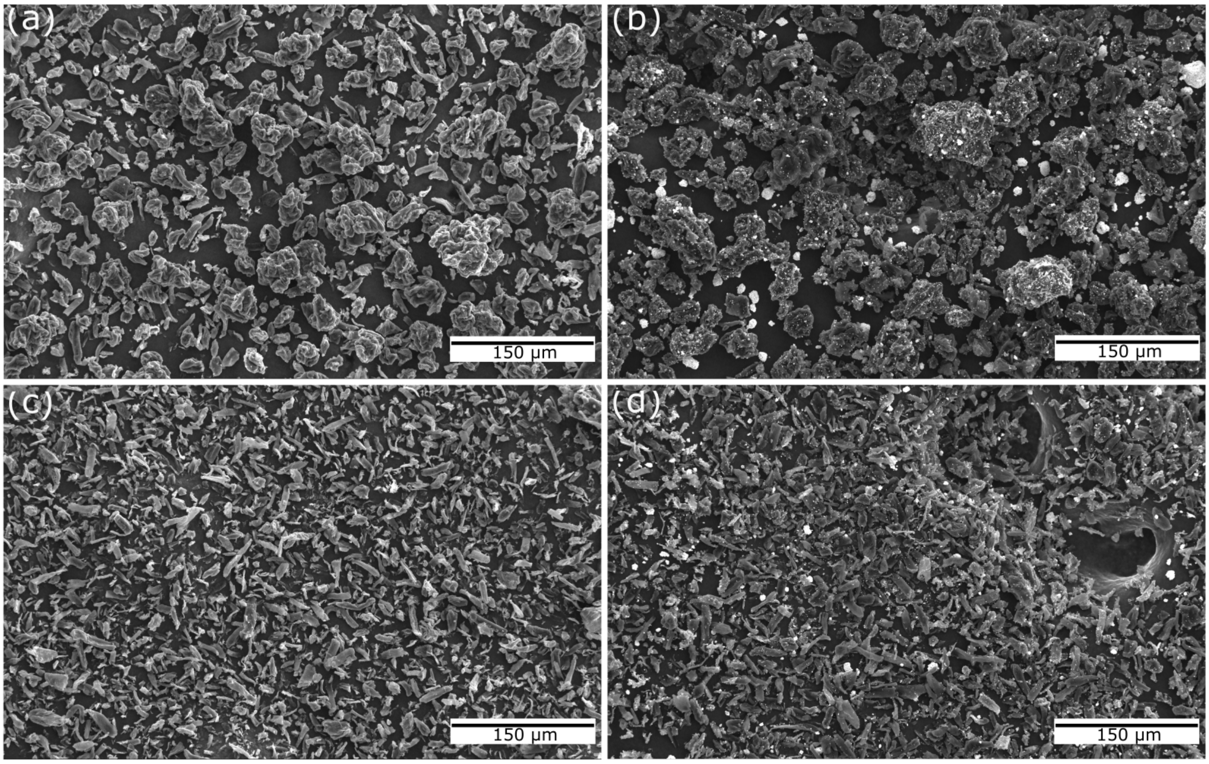

Figure S1: *low magnification SEM images of (a) CC, (b) CC-Sn, (c) FC and (d) FC-Sn*.

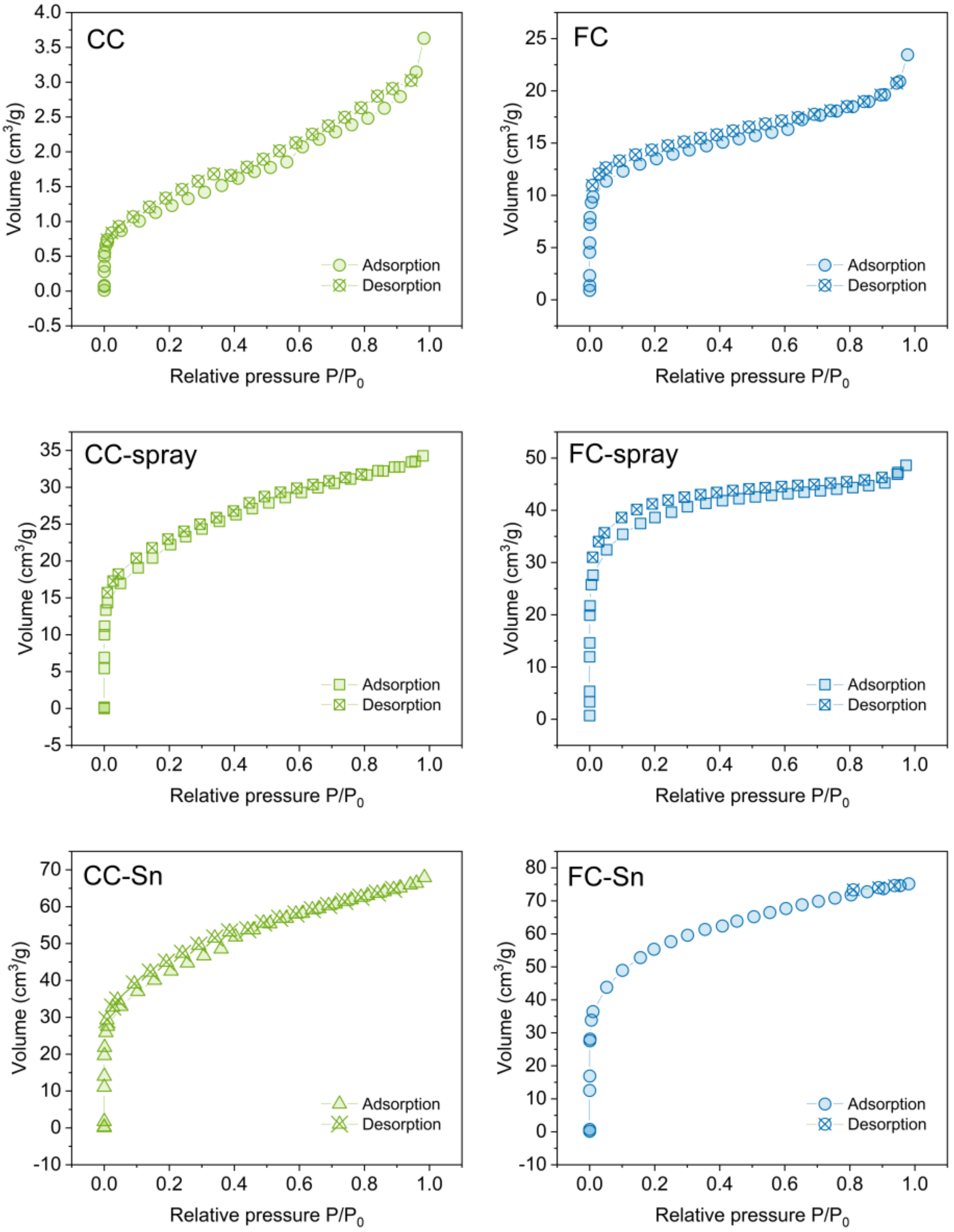

Figure S2: *BET volume profiles during N_2 gas adsorption and desorption*.

Material	*Surface area (m^2/g)*
CC	4
CC-spray	77
CC-Sn	148
FC	48
FC-spray	139
FC-Sn	195

Table S1: *N_2 BET measurement of the HC samples as synthesized (CC, FC), after the spray-dry process without Sn addition (CC-spray, FC-spray) and for the full composites prepared by spray-dry (CC-Sn, FC-Sn).*

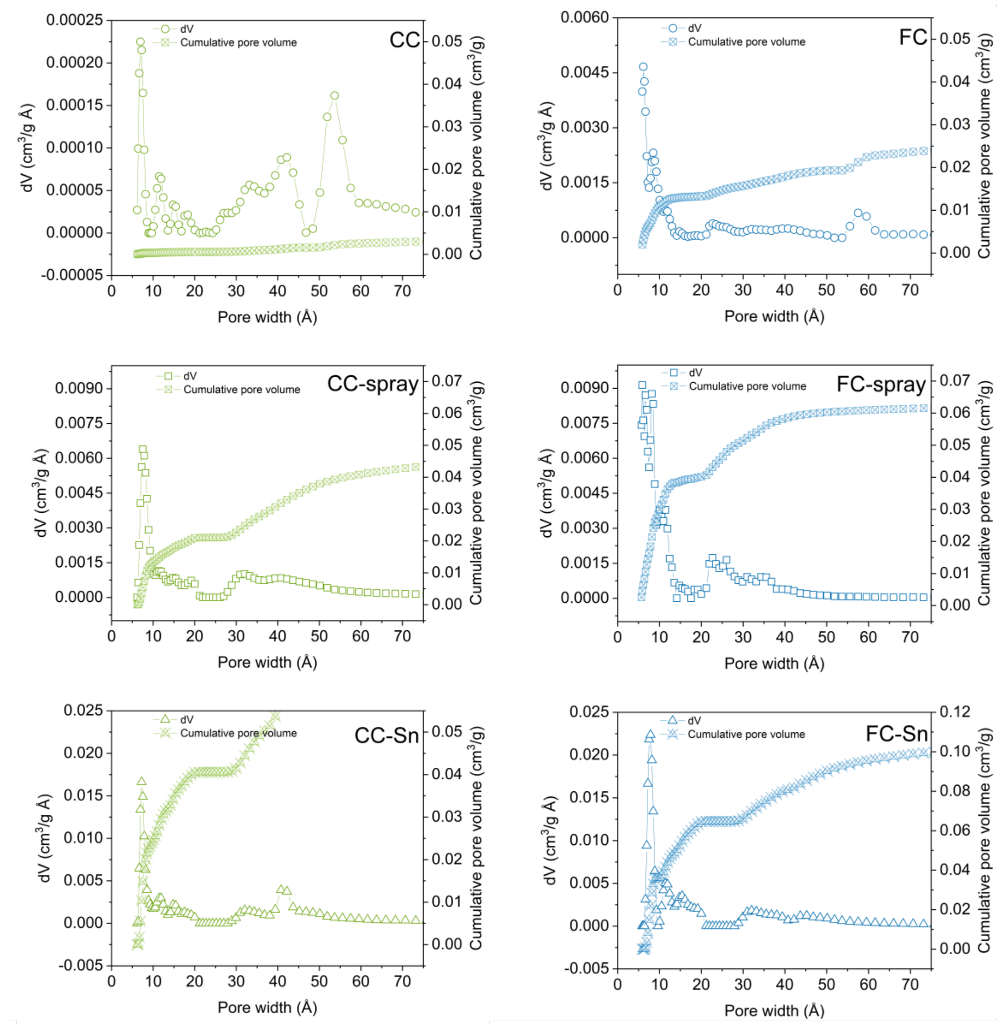

Figure S3: *BET pore size distribution obtained with DFT method.*

Material	*Pore width (Å)*
CC	6.940 ± 0.020
CC-spray	7.530 ± 0.033
CC-Sn	7.23 ± 0.024
FC	6.140 ± 0.008
FC-spray	5.900 ± 0.004
FC-Sn	7.850 ± 0.035

Table S2: *BET pore width obtained with DFT method.*

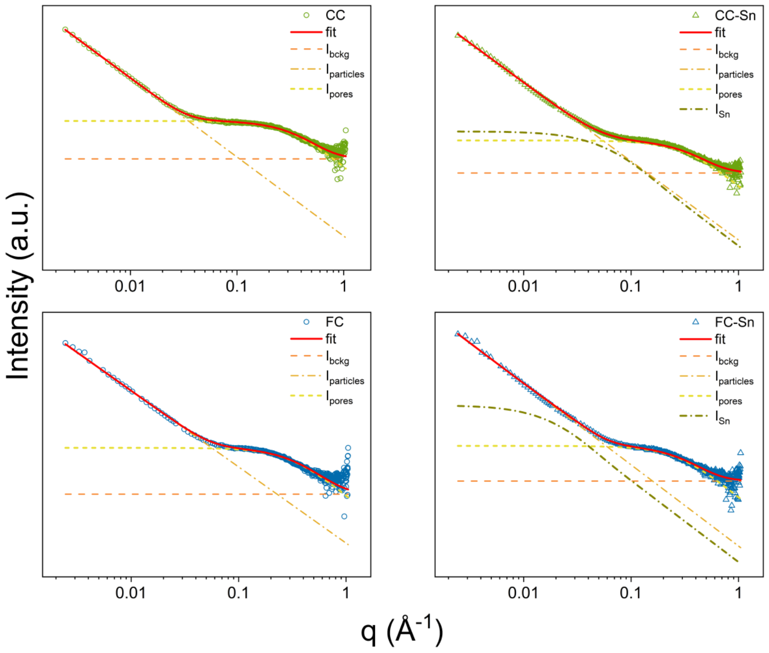

Figure S4: *SAXS data analysis. An extra contribution has been included for the HC-Sn composites.*

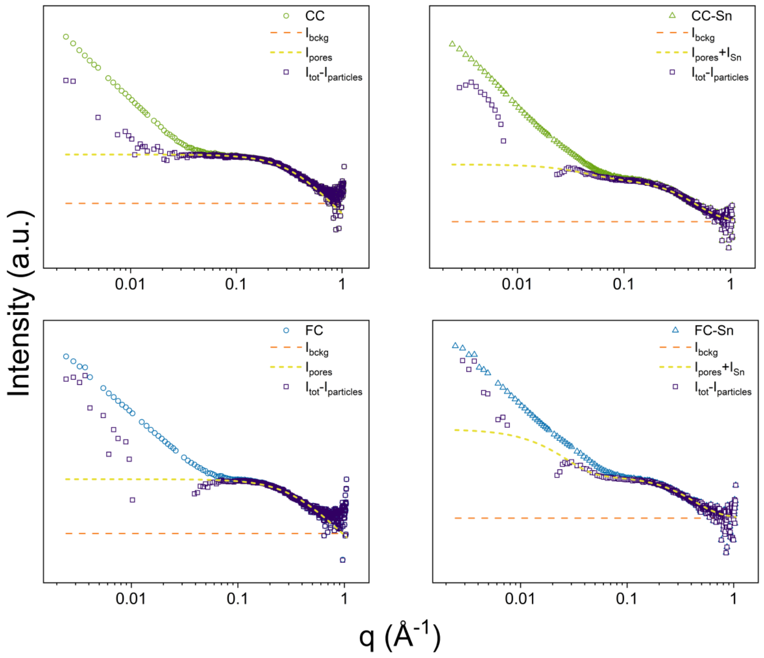

Figure S5: *SAXS data analysis subtracting the contribution of the particles. In the composites, the contribution of the pores alone is not enough to fit the experimental data.*

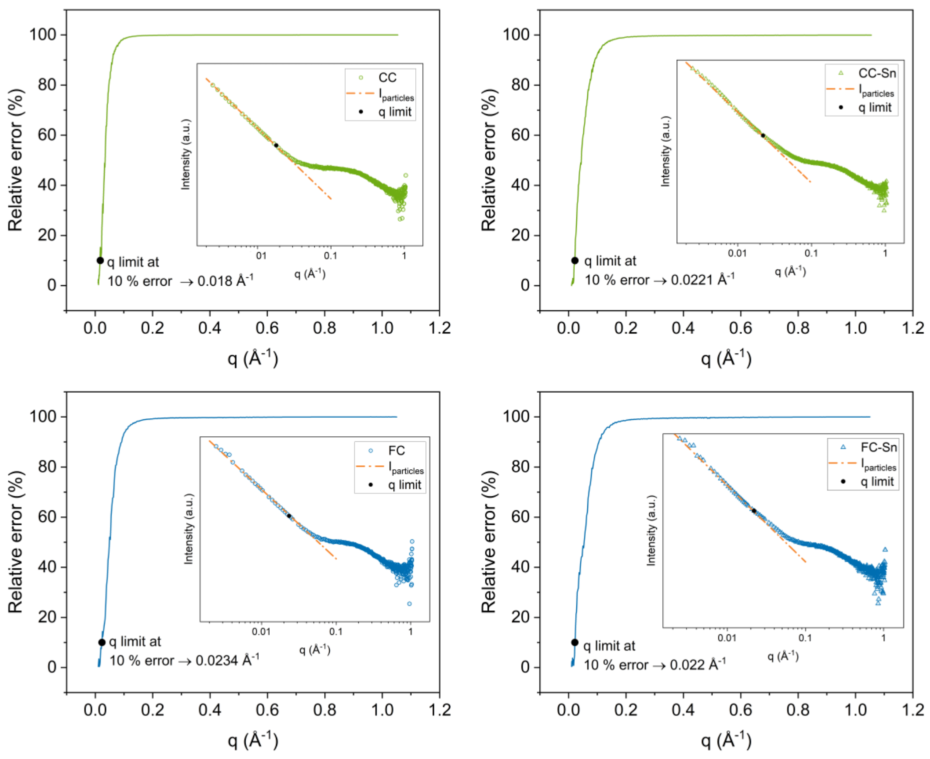

Figure S6: *extrapolation of q limit value by fitting the region at low q with the function $I_{particles}$. When the mismatching among the data and the function gets 10 %, the q value is taken.*

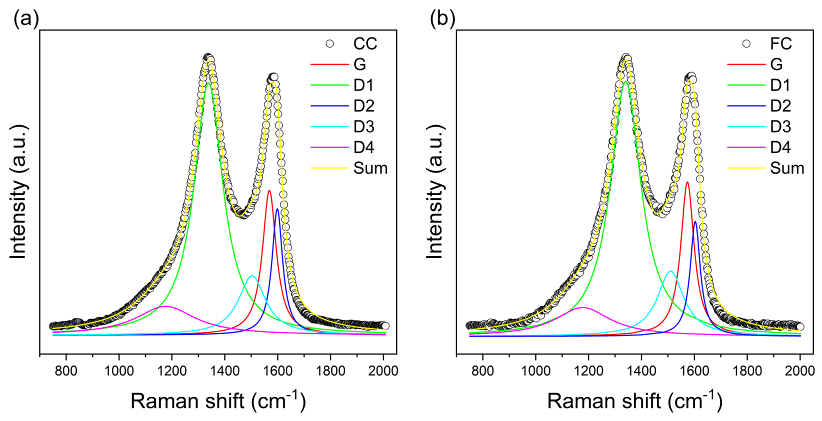

Figure S7: *Raman spectra of (a) CC and (b) FC. The Sadezky model has been used for the deconvolution.*

Equation	$y = y_0 + \frac{2A}{\pi}\left(\frac{w}{4(x-x_c)^2+w^2}\right)$				
**CC**					
	**G**	**D1**	**D2**	**D3**	**D4**
**y_0**	-0.02968 ± 0.00143	-0.02968 ± 0.00143	-0.02968 ± 0.00143	-0.02968 ± 0.00143	-0.02968 ± 0.00143
**x_c**	1568.68058 ± 1.84161	1338.46368 ± 0.29011	1598.59979 ± 1.21527	1502.88627 ± 4.41446	1175.95176 ± 6.36307
**w**	63.63411 ± 6.24186	133.21448 ± 1.60238	49.21425 ± 3.02301	124.8876 ± 10.20949	272.80376 ± 15.9402
**A**	53.53336 ± 11.6333	195.495 ± 3.42435	36.12998 ± 7.01352	43.12671 ± 7.00935	45.71952 ± 3.93176
**FC**					
**y_0**	-0.03444± 0.00174	-0.03444± 0.00174	-0.03444± 0.00174	-0.03444± 0.00174	-0.03444± 0.00174
**x_c**	1573.71273± 1.94711	1340.04061± 0.385	1510.20086± 4.92035	1177.54853± 7.41268	1603.31384± 1.50415
**w**	61.25609± 6.91881	141.36028± 2.11047	121.54407± 11.15478	252.89132± 18.84041	46.60664± 3.86398
**A**	55.01106± 13.28667	209.73478± 4.4729	46.14995± 8.43564	42.50393± 4.70721	31.11578± 7.76265

Table S3: *parameters used for fitting the spectra in Fig S7.*

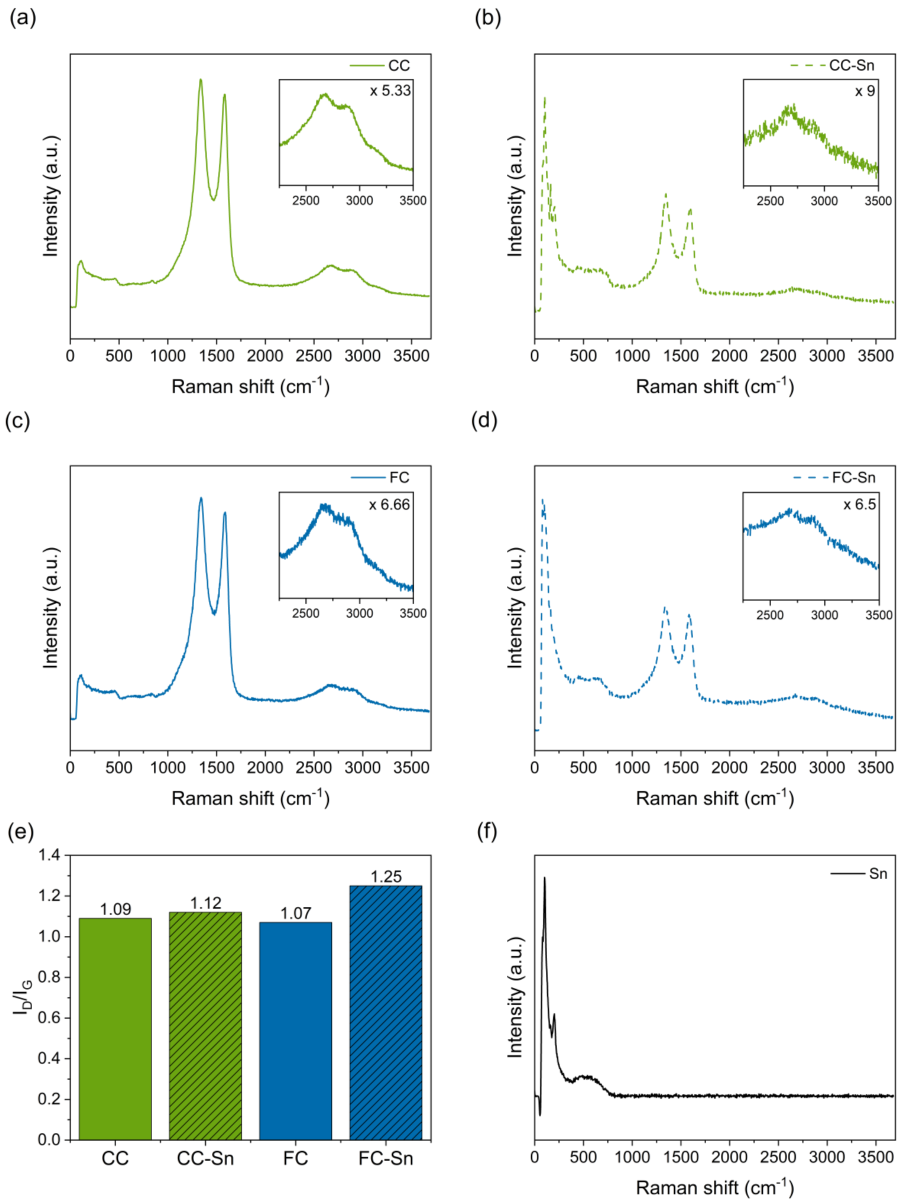

Figure S8: *Raman spectra of (a) CC, (b) CC-Sn, (c) FC, (d) FC-Sn. The degree of defectiveness of the samples has been determined by (e) I_D/I_G ratio. (f) Raman spectrum of Sn.*

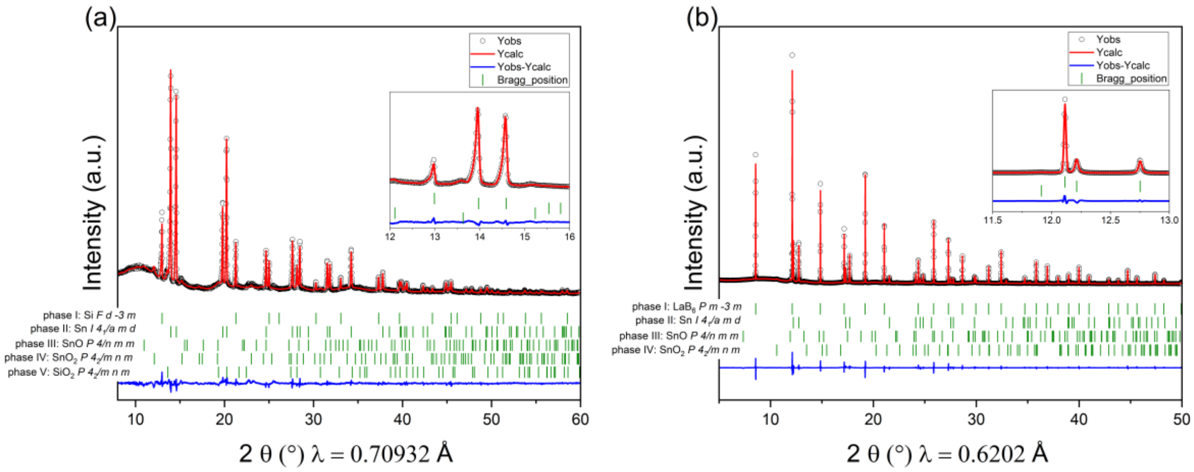

Figure S9: *Diffraction patterns of CC-Sn and reference in different ratios: (a) 10:1 with Si, (b) 15:2 with LaB_6. Pattern (a) was taken in lab in capillary (XRD), pattern (b) at the beamline MSPD in a capillary (SXRPD).*

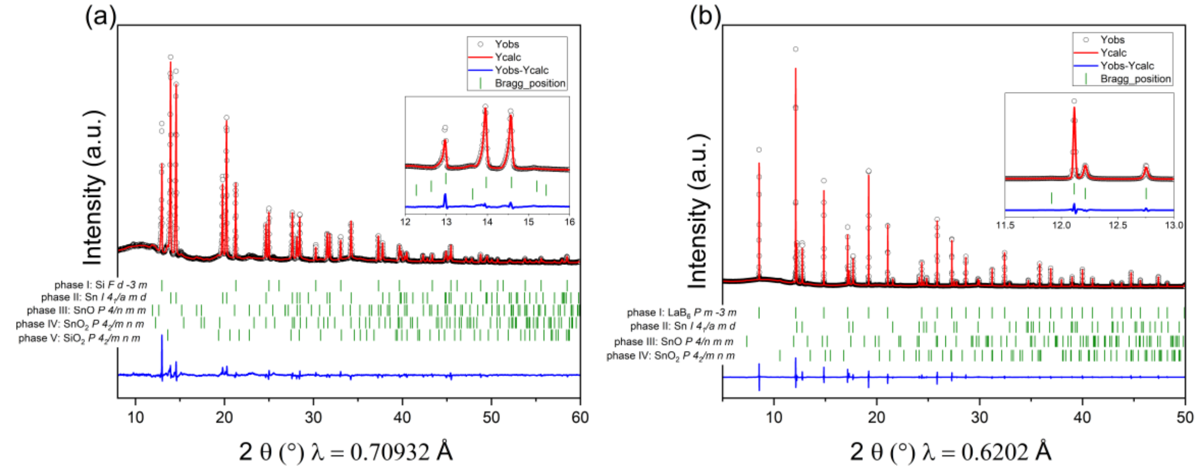

Figure S10: *Diffraction patterns of FC-Sn and reference in different ratios: (a) 10:1 with Si, (b) 15:2 with LaB_6. Pattern (a) was taken in lab in capillary (XRD), pattern (b) at the beamline MSPD in a capillary (SXRPD).*

Material	reference	λ (Å)	AM : reference	Amorphous content (%)	R_{Bragg} reference (%)	R_{Bragg} Sn (%)
CC-Sn	LaB_6	0.62020	15 : 2	93.27 ± 0.06	2.77	7.68
FC-Sn	LaB_6	0.62020	15 : 2	93.37 ± 0.08	3.17	6.3

Table S4: *data of XRD measurements and refinements with LaB_6 to quantify the amorphous phase in the composites.*

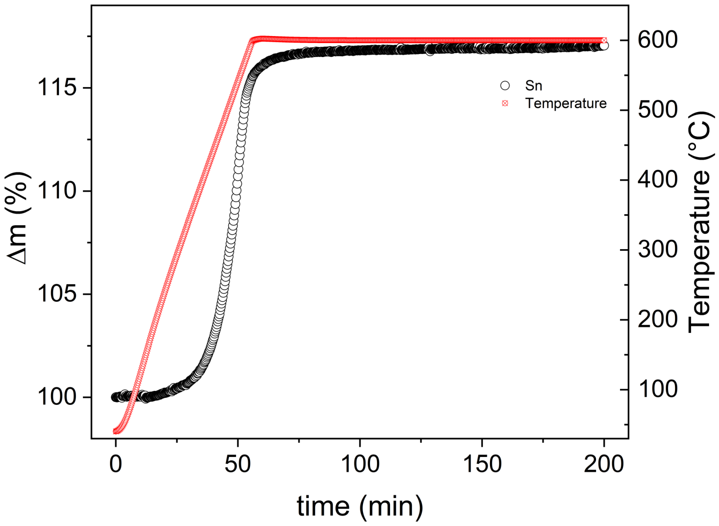

Figure S11: *TGA measurement of Sn in air. At the end of the experiment, SnO_x has formed (1<x<2 based on the experimental value).*

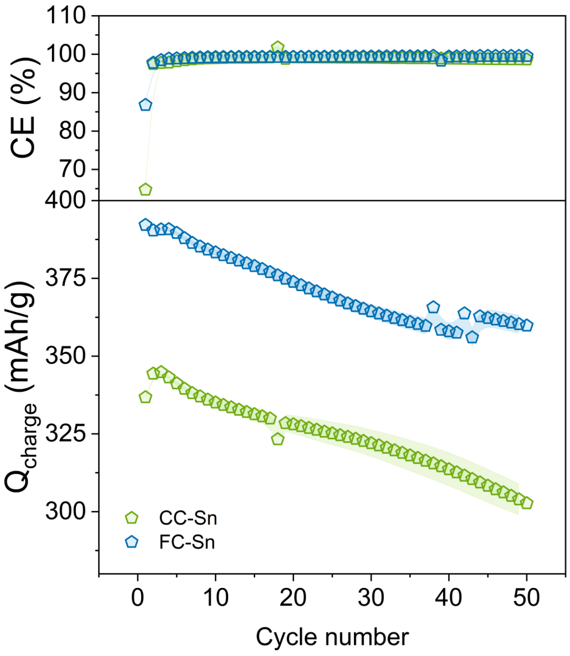

Figure S12: *Gravimetric capacity (bottom) and coulombic efficiency (top) of CC-Sn and FC-Sn during electrochemical cycling in $NaPF_6$ 1 M in diglyme electrolyte.*

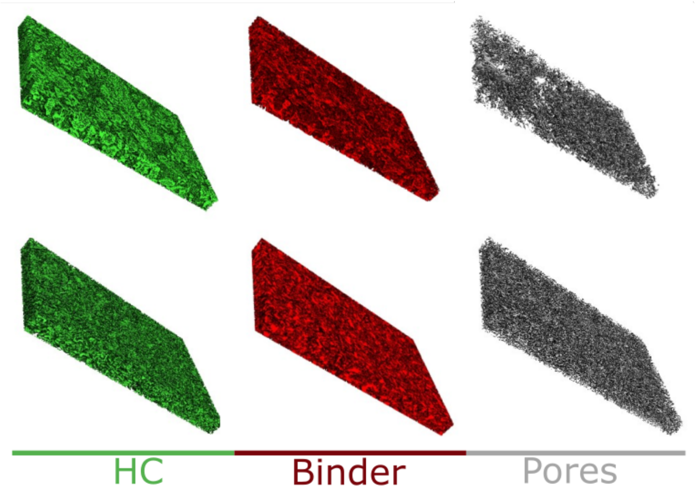

Figure S13: *3D XRM reconstruction of a section of HC electrodes (CC on top and FC on the bottom).*

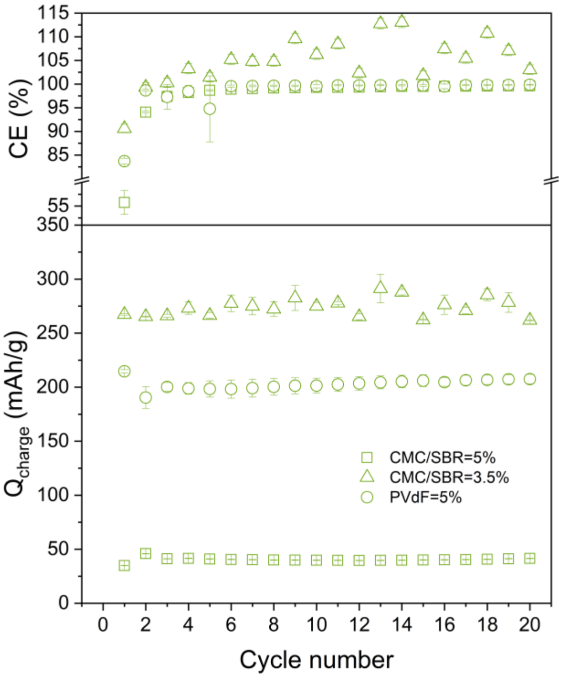

Figure S14: *capacity retention @C/10 (bottom) and CE (top) of CC with different binders: PVDF 5 % (circle), CMC/SBR 3.5 % (triangle) and CMC/SBR 5 % (square). Testing is performed in half cells at 25 °C in the voltage window 0.002-2 V vs. Na^{+}/Na.*

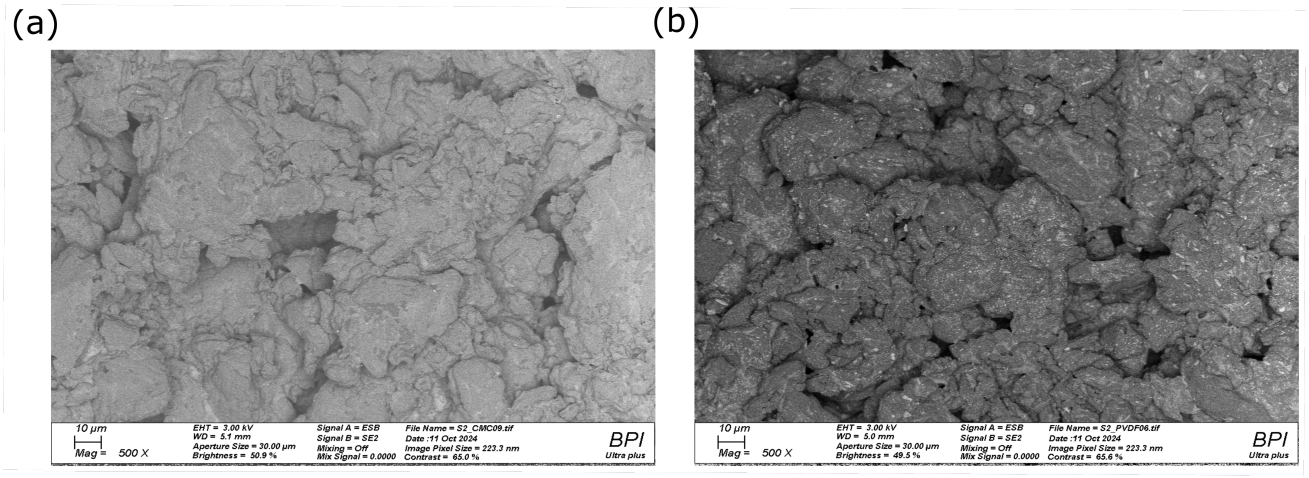

Figure S15: *comparison of CC electrodes prepared with (a) CMC/SBR and (b) PVdF. CMC:SBR binder gives a thick insulating layer that obstacles the sodium insertion in the active material.*

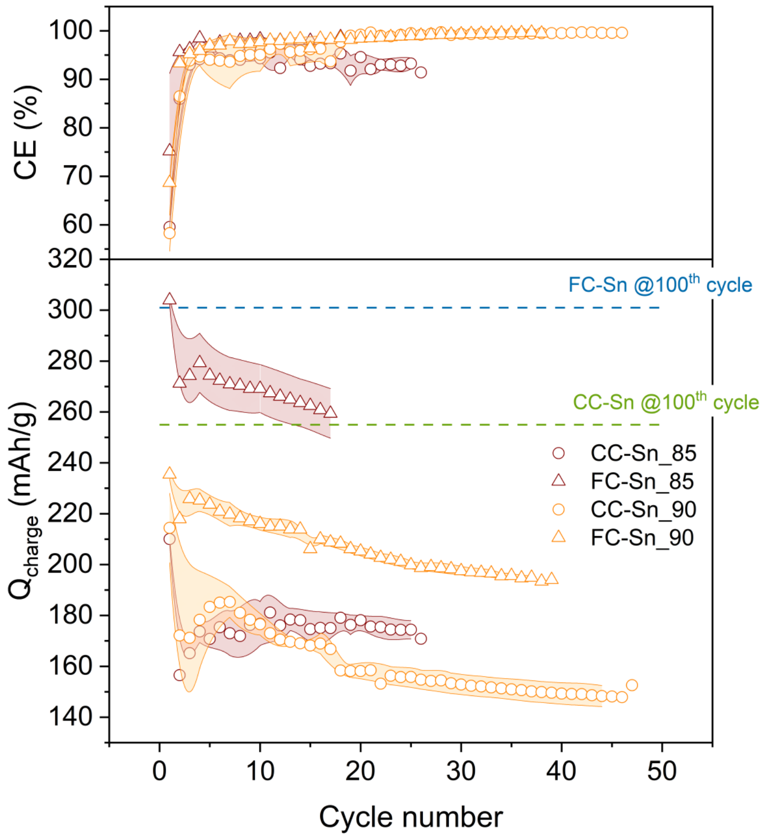

Figure S16: *Gravimetric capacity vs cycle number (bottom) and coulombic efficiency (top) of CC-Sn and FC-Sn @C/10 with different active material percentage in the electrode formulation: 85 % for CC-Sn_85 and FC-Sn_85 and 90% for CC-Sn_90 and FC-Sn_90. The horizontal lines indicate the gravimetric capacity of the reference composites CC-Sn (with PVDF) and FC-Sn after 100 cycles.*

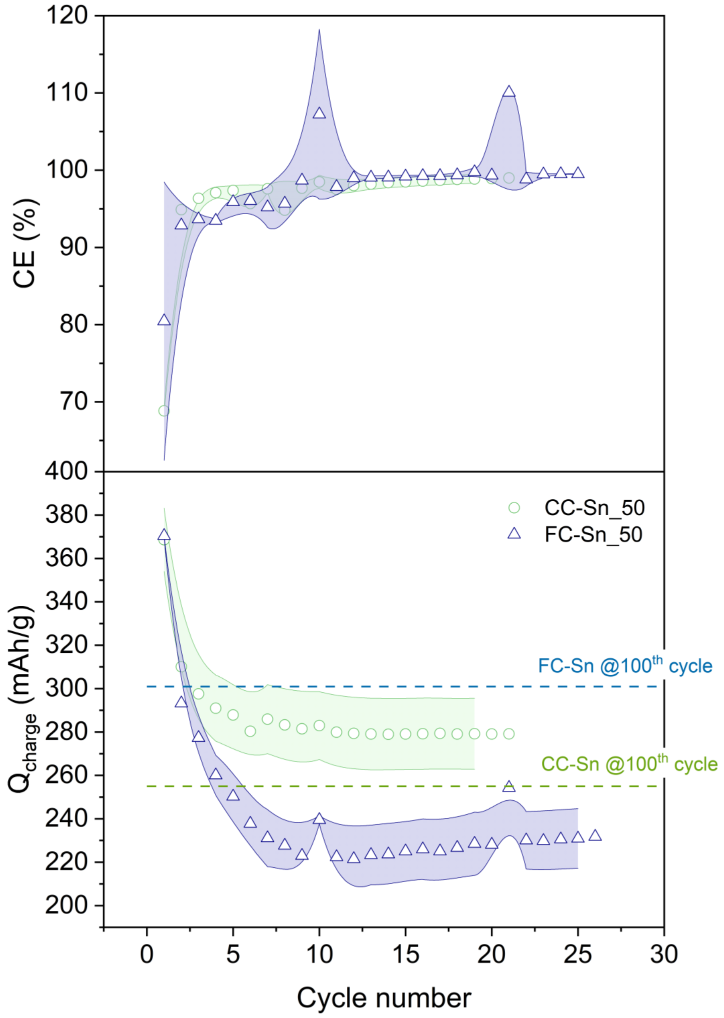

Figure S17: *Gravimetric capacity vs cycle number (bottom) and coulombic efficiency (top) of CC-Sn_50 and FC-Sn_50 @C/10. The horizontal lines indicate the gravimetric capacity of the reference composites CC-Sn and FC-Sn after 100 cycles.*

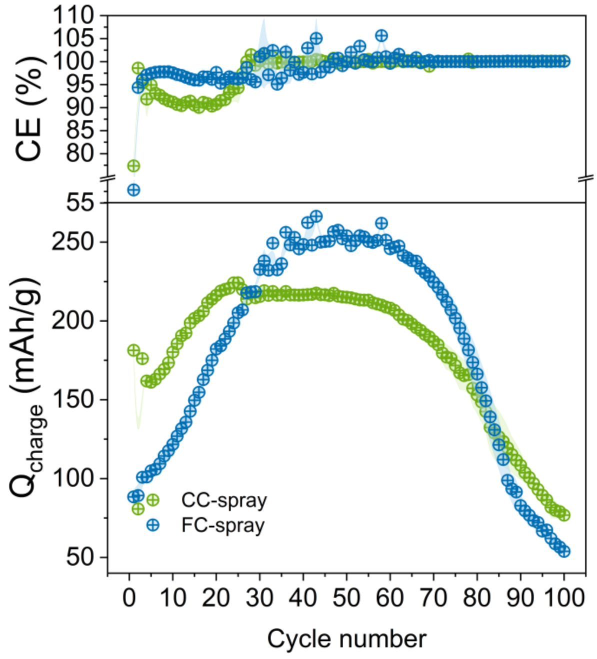

Figure S18: *Gravimetric capacity vs cycle number (bottom) and coulombic efficiency (top) of CC and FC hard carbons after the spray drying step performed without Sn.*

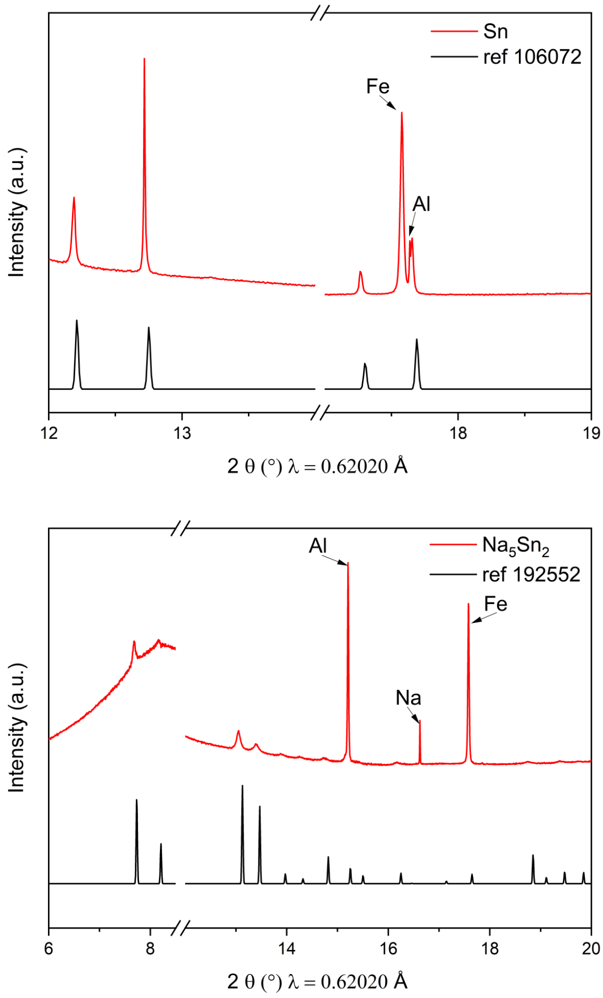

Figure S19: *first (top) and last (bottom) sXRD patterns of CC-Sn at 50 °C during electrochemical discharge. We also report the references for phase identification from the ICSD database.*

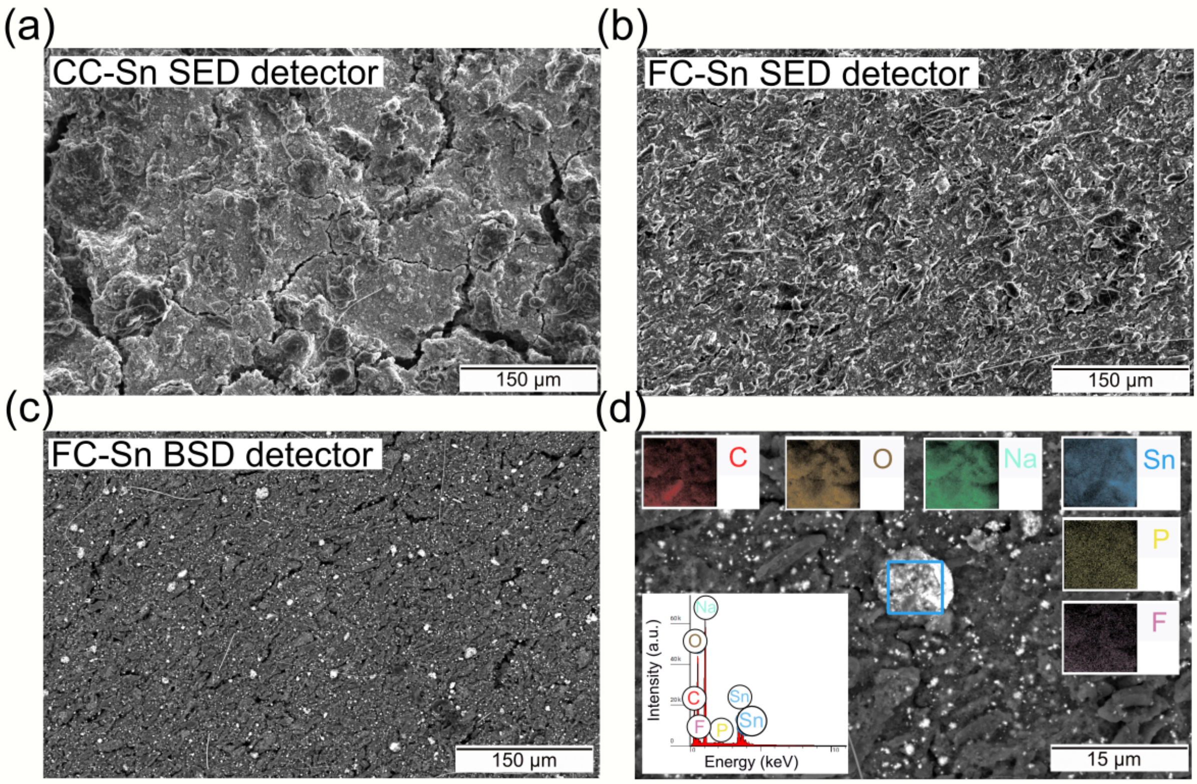

Figure S20: *ex situ SEM at end of electrochemical discharge of (a) CC-Sn and (b) FC-Sn with SED detector. (c) Ex situ SEM at end of electrochemical discharge of FC-Sn with BSD detector and (d) its EDX analysis. The elemental composition of the Na-Sn alloy particle is highlighted, and the spectrum is reported and fitted.*

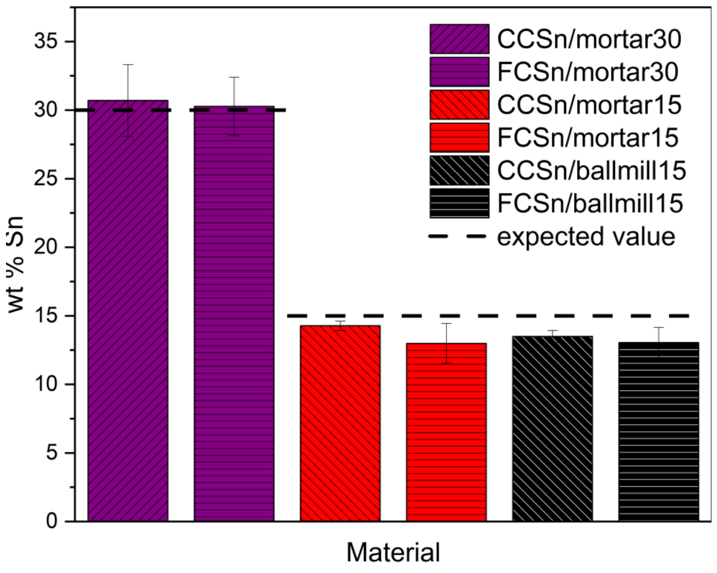

Figure S21: *Sn wt% determination with TGA of the materials used as comparison of spray dried samples. The dash line indicates the expected Sn wt% in the samples.*

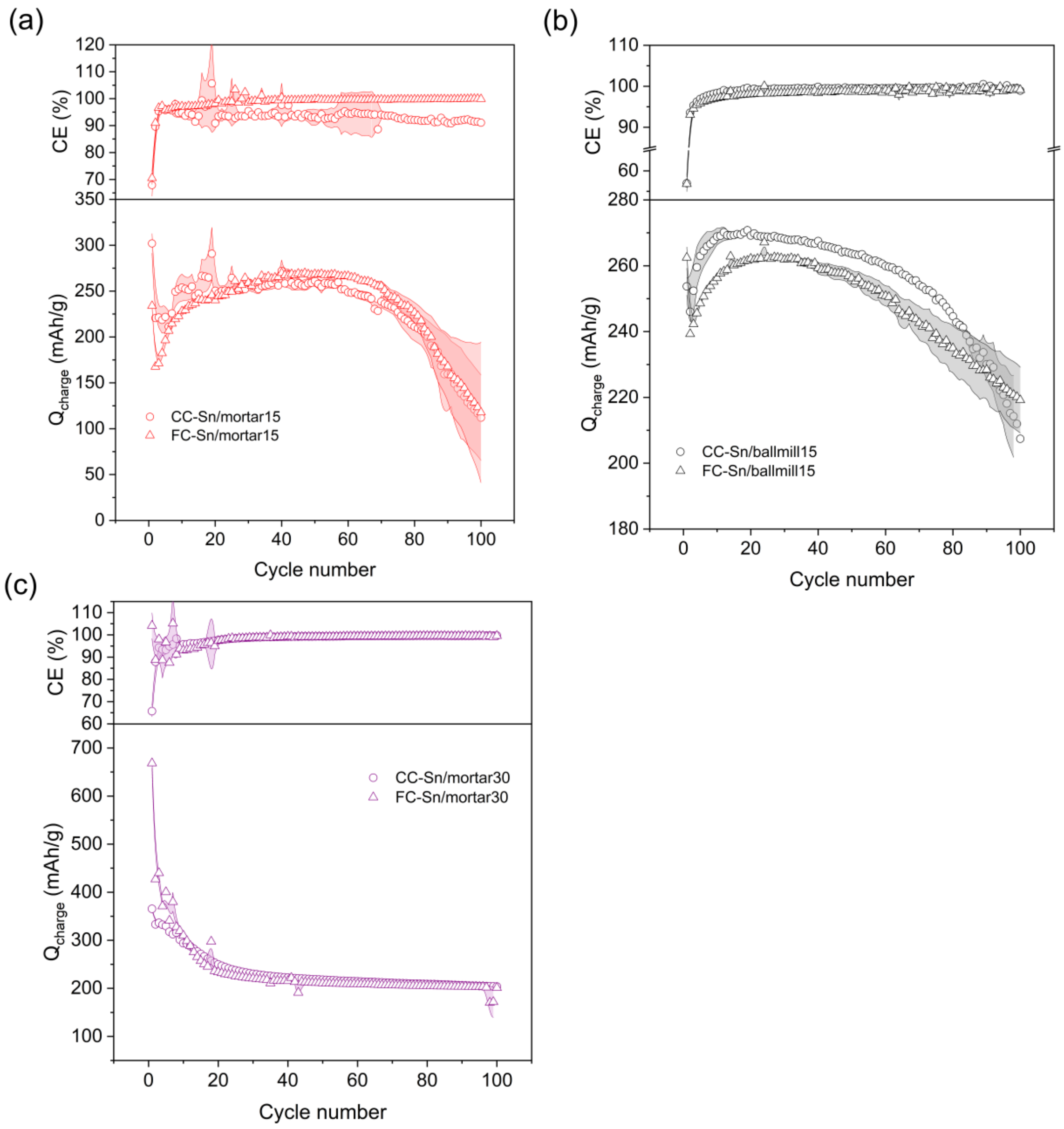

Figure S22: *gravimetric capacity vs cycle number (bottom) and CE (top) of: (a) samples prepared in mortar with 15 wt% Sn, (b) samples prepared in ball milling jars with 15 wt% Sn, (c) samples prepared in mortar with 30 wt% Sn.*
